# Symmetric resonator based tunable epsilon negative near zero index metamaterial with high effective medium ratio for multiband wireless applications

**DOI:** 10.1038/s41598-021-01266-7

**Published:** 2021-11-08

**Authors:** Md. Moniruzzaman, Mohammad Tariqul Islam, Ismail Hossain, Mohamed S. Soliman, Md Samsuzzaman, Sami H. A. Almalki

**Affiliations:** 1 Department of Electrical, Electronic and Systems Engineering, Faculty of Engineering and Built Environment, Universiti Kebangsaan Malaysia, Bangi, Malaysia; 2 Space Science Center (ANGKASA), Universiti Kebangsaan Malaysia, 43600 UKM Bangi, Selangor Malaysia; 3 Department of Electrical Engineering, College of Engineering, Taif University, P.O. Box 11099, Taif, 21944 Kingdom of Saudi Arabia; 4 Department of Electrical Engineering, Faculty of Energy Engineering, Aswan University, Aswân, 81528 Egypt; 5 Department of Computer and Communication Engineering, Faculty of Computer Science and Engineering, Patuakhali Science and Technology University, Patuakhali, Bangladesh

**Keywords:** Electrical and electronic engineering, Metamaterials

## Abstract

In this paper, a tuned metamaterial (MTM) consisting of a symmetric split ring resonator is presented that exhibits epsilon negative (ENG), near zero permeability and refractive index properties for multiband microwave applications. The proposed metamaterial is constituted on a Rogers (RT-5880) substrate with 1.57 mm thickness and the electrical dimension of 0.14λ × 0.14λ, where wavelength, λ is calculated at 4.2 GHz. The symmetric resonating patch is subdivided into four equal and similar quartiles with two interconnecting split rings in each quartile. The quartiles are connected at the center of the substrate with a square metal strip with which four tuning metal strips are attached. These tuning metal strips are acted as spacers between four quartiles of the resonator patch. Numerical simulation of the proposed design is executed in CST microwave studio. The proposed MTM provides four resonances of transmission coefficient (S_21_) at 4.20 GHz, 10.14 GHz, 13.15 GHz, and 17.1 GHz covering C, X and Ku bands with negative permittivity, near zero permeability and refractive index. The calculated effective medium ratio (EMR) is 7.14 at 4.2 GHz indicates its compactness. The resonance frequencies are selective in nature which can be easily tuned by varying the length of the tuning metal stubs. The equivalent circuit of the proposed MTM is modelled in Advanced Design Software (ADS) that exhibits a similar S_21_ compared with CST simulation. Surface current, electric and magnetic fields are analyzed to explain the frequency tuning property and other performances of the MTM. Compact size, ENG with near zero permeability and refractive index along with frequency selectivity through tuning provides flexibility for frequency selective applications of this MTM in wireless communications.

## Introduction

Metamaterial as an artificial medium exhibits some exotic properties such as negative permittivity, negative permeability, negative refractive index, which makes it suitable for various applications such as absorber^1^, microwave imaging^2^, bio sensing^3^, antennas^4^, metamaterial lensing^5^, metamaterial coding^6^, terahertz metamaterial absorber^7–9^, and microwave devices like Bluetooth, WiMAX, GPS^5,10^. A negative index metamaterial is present in^11^ that consists of elongated beams attached to a plate. A 3D acoustic metamaterial is discussed in^12^ that is used to create a bandgap at the point of deep sound attenuation that can be used for noise cancellation as an acoustic filter. All the properties of these metamaterials have occurred at some fixed frequencies of interest depending on the particular geometrical arrangement in the array and with the fixed composition of the structure. Therefore, there is a growing interest on metamaterial focusing different working frequencies those will be tuned by various stimulus such as electrical, mechanical, or optical. Ou et al. present a metamaterial made with Au layer on Si substrate and operating in the terahertz range. This terahertz MTM exhibits tunable characteristics to adjust resonances with the manipulation of switching windows^13^. A magnetically tuned metamaterial is presented in^14^, in which isotopic dielectric ferric cylinders are periodically hosted. These ferric rods are partially magnetized by applying a dc voltage that makes the real part of the magnetic field varying negative to positive values. In^15^, a multilayered hyperbolic metamaterial is presented for controlling charge transfer dynamics. An electrically controllable metamaterial is presented in^16^ that can be used for the topological transition of an iso-frequency contour^16^.

A metamaterial-based absorber is presented in^17^ that is based on concentric interconnected split ring resonator on FR4 substrate. This metamaterial is asymmetrical in structure, and 97.9%, 99.5%, 99.1% and 99.95% absorption peaks are obtained at 4.1 GHz, 11.3 GHz, 6.86 GHz, and 13.45 GHz, respectively. A metamaterial consisting of an S-shaped resonator is designed by Sabah et al*.* that is operated in L band for application in microwave sensing^18^. A cross-coupled resonator-based MTM is presented in^19^ that exhibits negative permittivity and near zero refractive index with 8.03 EMR for satellite and RADAR communications. In addition, in^20–23^, some metamaterials and resonators are presented for various applications and property analysis. A Gap coupled hexagonal split-ring resonator-based metamaterial with 10 × 10 mm^2^ dimension covering S and X band is explained in^24^. On the other hand, metamaterial with the concentric ring-based resonator is demonstrated in^25^ that exhibits single negative property with two resonances at 13.9 GHz, 27.5 GHz to enhance the performance of the microstrip transmission line. An open delta shaped ENG metamaterial is reported in^26^ that provides a triple-band response. In addition, a complementary split-ring resonator (CSRR) with pi-shaped metal inclusion is designed and presented in^27^ for S, C, and X-bands microwave applications. In^28^, the metamaterial is utilized to reduce mutual coupling and bandwidth enhancement of MIMO antenna, whereas terahertz band pass filter is reported in^29^ using metamaterial based photonic structure. In another literature, terahertz metamaterial is used as a filter with tuning capability within the frequency range from 0.53 THz to 0.76 THz covering optical windows for indoor wireless communications^30^. Moreover, another tunable metamaterial is reported in^31^, where MTM nanodisks are placed on Si substrate and with elevating the column number of the MTM nanodisks, transmission intensity modulation is realized. MA Bakir demonstrates a metamaterial absorber based on gallium-doped zinc oxide (GZO) nanowire that exhibits wide band absorption in near infrared regime (NIR) and short-wavelength infrared regime (SWIR)^32^. In another article, a metamaterial absorber is presented that is constructed with tungsten film coated over silicon oxide (SiO_2_). Absorptivity of this MTM depends on the nanohole radius of tungsten^33^. Bilal et al. presents a metamaterial absorber operating in visible regime in which elliptical shaped tungsten metal rings are used providing an average absorption of 90%^34^.

In this paper, Symmetric resonator-based tuned metamaterial is presented that shows four resonances of S_21_ covering C, X and Ku bands. The novelty of this design is that the MTM's resonator patch can be divided into four elements that are mirror-symmetric to each other. The resonance frequency of S_21_ can be controlled through the change of length of four tuning metallic stubs those act as a spacer between four quartiles of the resonating patch. Thus, this MTM structure can be used for different frequency applications by tuning to appropriate frequencies. Moreover, this MTM provides negative permittivity, near zero permeability and near zero refractive index with a good EMR value. The negative permittivity property of the MTM helps to enhance antenna bandwidth^35^, filter design^36^, whereas near-zero index helps to enhance antenna gain and directivity^37–39^. So, this MTM can be applicable to various microwave devices in wireless communications. The rest of the manuscript is organized as follows: metamaterial unit cell design, simulation method and equivalent circuit modelling is performed in “Metamaterial (MTM) unit cell design, simulation and equivalent circuit modelling” section. Frequency tuning property is discussed in “Frequency tuning of proposed MTM” section with the electric field, magnetic field and surface current analysis. In “Result and analysis” section result analysis and discussion are made that includes analyses of various properties of MTM such as permittivity, permeability, refractive index, impedance and power associated with MTM along with the electric field, surface current and magnetic field study. The measurement result is also discussed in this section. Moreover, this section also includes a comparison of the proposed MTM performance with other states of arts. Finally, the conclusion is made in “Conclusion” section, highlighting the major outcomes of the proposed design.

## Metamaterial (MTM) unit cell design, simulation and equivalent circuit modelling

### Unit cell design and simulation

**Figure 1:**
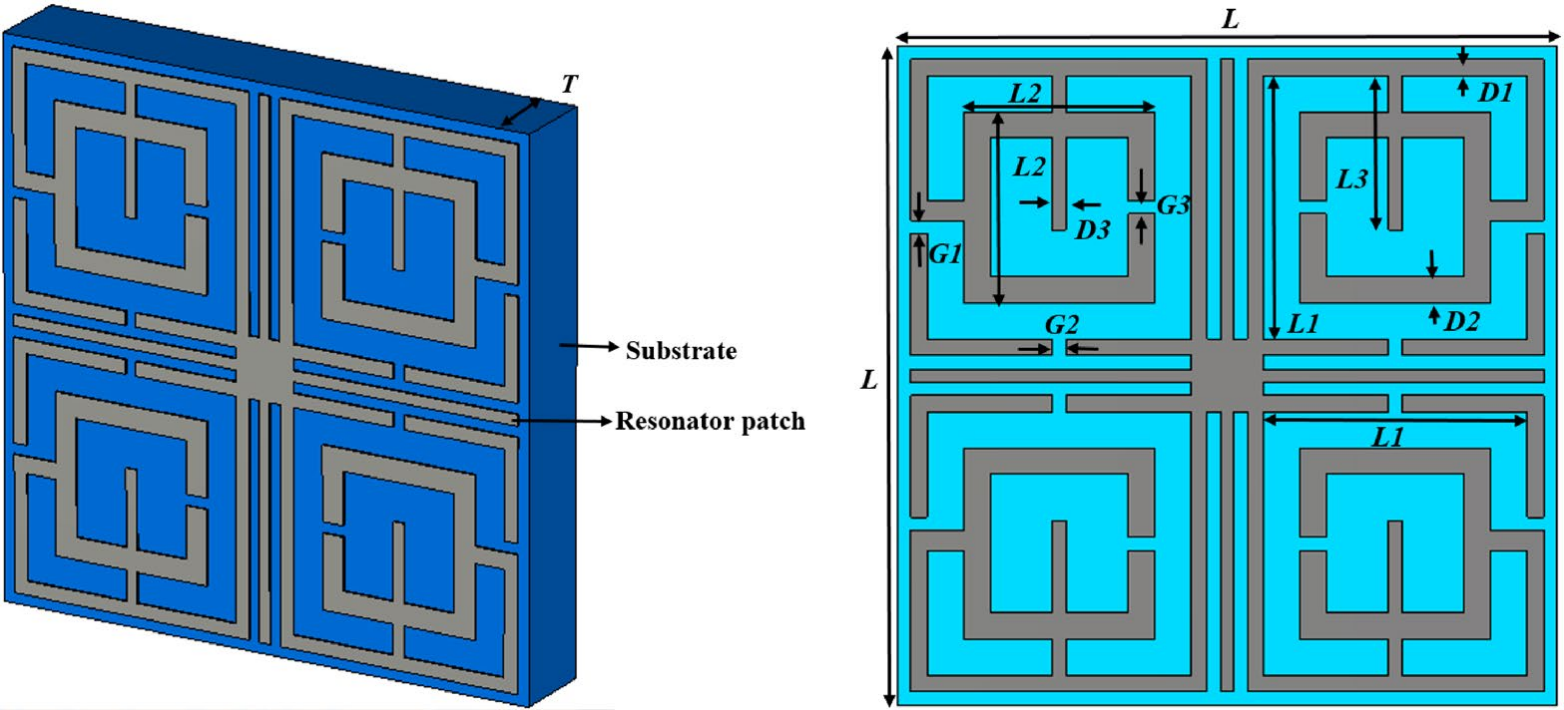
(**a**) Proposed MTM unit cell. (**b**) Structural layout of the patch of the proposed unit cell (CST STUDIO SUITE 2019, https://www.3ds.com/products-services/simulia/products/cst-studio-suite)^42^.

The proposed MTM unit cell is designed on Rogers RT 5880 substrate having a thickness of 1.57, dielectric constant 2.2 and loss tangent of 0.0004 and it is composed of glass microfiber reinforce PTFE. It exhibits uniform electrical properties over wide frequency range with low moisture absorption that makes it suitable for high moisture environments. Moreover, its lowest electrical loss makes it suitable for high frequency applications. Since in this work, operating frequency bands are extended from 4 GHz to 18 GHz, so Rogers RT5880 is used that provides good performance in the high frequency regions. The dimension of the substrate is selected 10 × 10 mm^2^ that makes the unit cell small enough compared to wavelength in our target frequency ranges from 4 to 18 GHz covering C, X and Ku-bands so that effective response of the metamaterial can be realized^40^. The resonating patch is constructed on this substrate material with conductor metal strips having a thickness of 0.035 mm. The patch is a twofold mirror-symmetric structure that can be divided into four equal parts. Each part contains two square split-ring resonators that are connected by two metal strips. The quartiles are then interconnected by squared shaped metal strip placed at the center of the total structure. Four tuning metallic stubs are extended towards the two perpendicular axes from the bisecting points of four sides of this central squared metal strip. The length of these metallic stubs plays a vital role in changing the frequency of resonances. The frequency of resonances can be changed with the help of modifying the length of these stubs. The complete MTM unit cell is presented in Fig. 1a, and the structural layout of the resonator side is presented in Fig. 1b. The various dimensions of different elements of the resonator patch along with substrate are presented in Table 1. The numerical simulation is performed in CST microwave studio suite 2019 using a frequency domain solver that utilizes the finite element method (FEM) for numerical analysis to solve electromagnetic field equations^41^. FEM explored two curl equations of Maxwell in three dimensional form to analyze three dimensional electromagnetic fields. Helmholtz equation to derive electric field intensity is formed using the curl equations of Maxwell and expressed as:

1 $$\Delta \times \left( {\frac{1}{{\mu_{r} }}\nabla \times E} \right) - k_{0}^{2} \varepsilon_{r} E_{ } = 0_{ }$$

here E(x,y,z) is the electric field distribution of the wave vector, $k_{0}$ is wave number in free space, $\mu_{r}$ and $\varepsilon_{r}$ are the permeability and permittivity of the medium. Using variational principal, Eq. (1) can be written as function having the form:

2 $$F\left( E \right) = \int {\int {\int\limits_{\theta } {\left\{ {\frac{1}{{\mu_{r} }}\left( {\nabla \times E} \right) \cdot \left( {\nabla \times {\text{E}}} \right) - k_{0}^{2} \varepsilon_{r} E \cdot E} \right\}d\theta } } } .$$

**Table 1:** Design parameters of proposed MTM unit cell.

Parameter	Dimension (mm)	Parameter	Dimension (mm)	Parameter	Dimension (mm)
*L*	10	*D1*	0.25	*G2*	0.2
*L1*	4	*D2*	0.4	*G3*	0.2
*L2*	2.9	*D3*	0.2	*T*	1.57
*L3*	2.35	*G1*	0.2		

**Figure 2:**
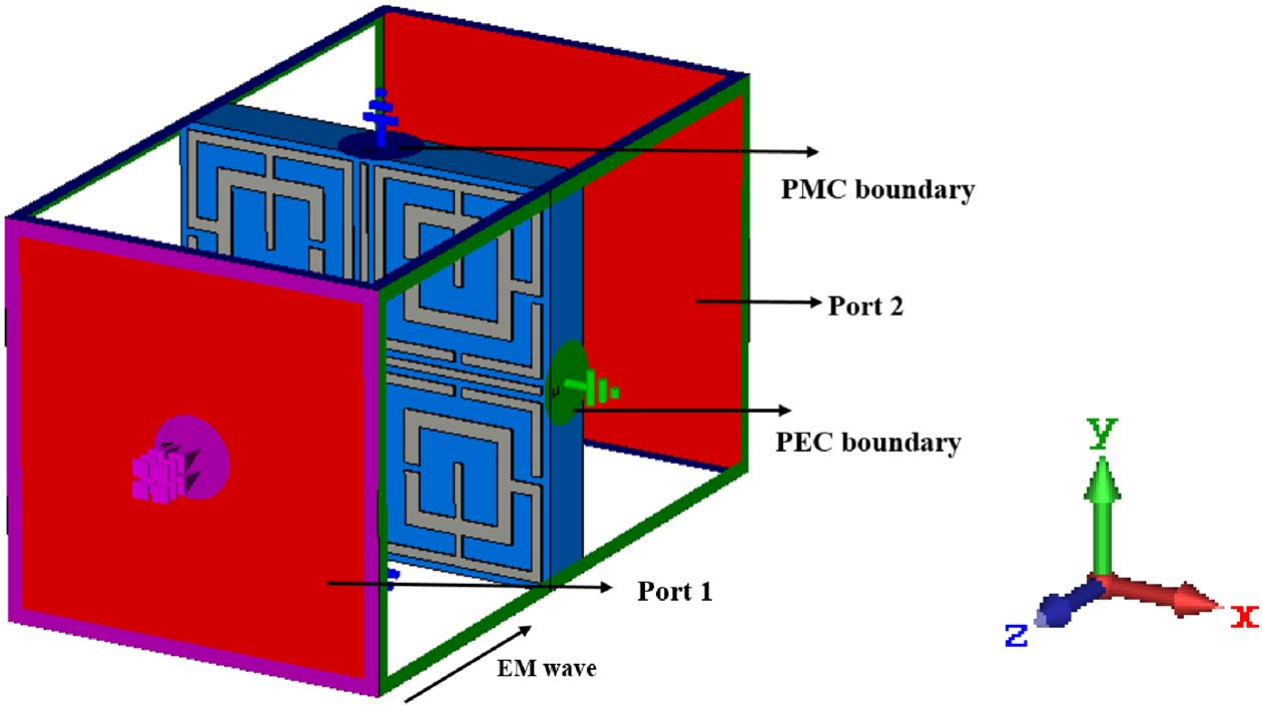
Simulation setup for performance evaluation of the unit cell (CST STUDIO SUITE 2019, https://www.3ds.com/products-services/simulia/products/cst-studio-suite)^42^.

FEM utilizes variation principle, and through numerical analysis, the solution is obtained by dividing the solution domain into different unit areas, applying the local function to each subdomain field. Then fields are associated by the nodes, and the entire field constructs grids of subdomain nodes. Now full wave solution of the problem is obtained S parameters in FEM by solving various equations relating the nodes, energy equations with certain precision and boundary constraint^41^. The simulation arrangement of the proposed MTM is depicted in Fig. 2, where the incident electromagnetic field is applied normal to the resonator in Z-axis, whereas perfect electrical conductor (PEC) and perfect magnetic conductor (PMC) boundaries are applied in X and Y axis, respectively. The simulation is performed from 2 to 18 GHz for the observation of the transmission coefficient (S_21_) and reflection coefficient (S_11_).

### Evolution steps of the proposed MTM unit cell

**Figure 3:**
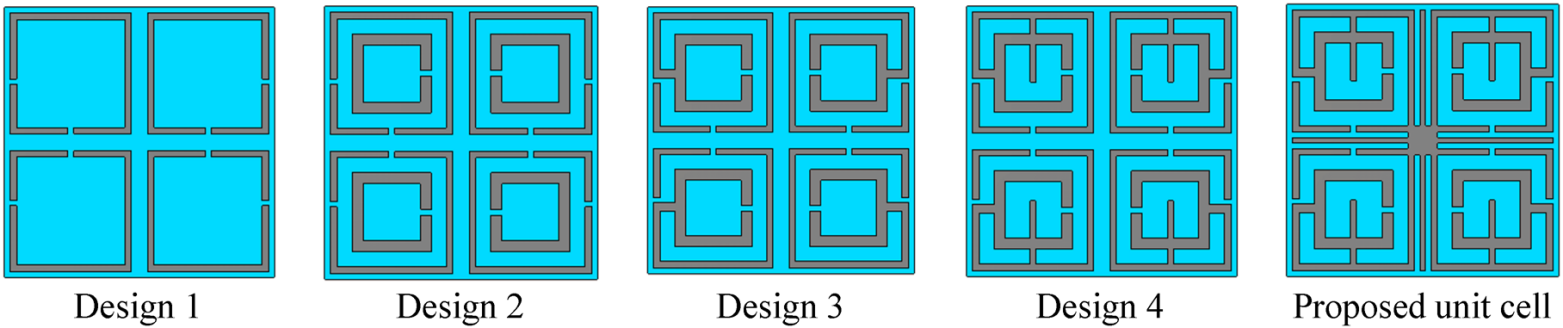
Different evolution steps towards the proposed unit cell (CST STUDIO SUITE 2019, https://www.3ds.com/products-services/simulia/products/cst-studio-suite)^42^.

**Figure 4:**
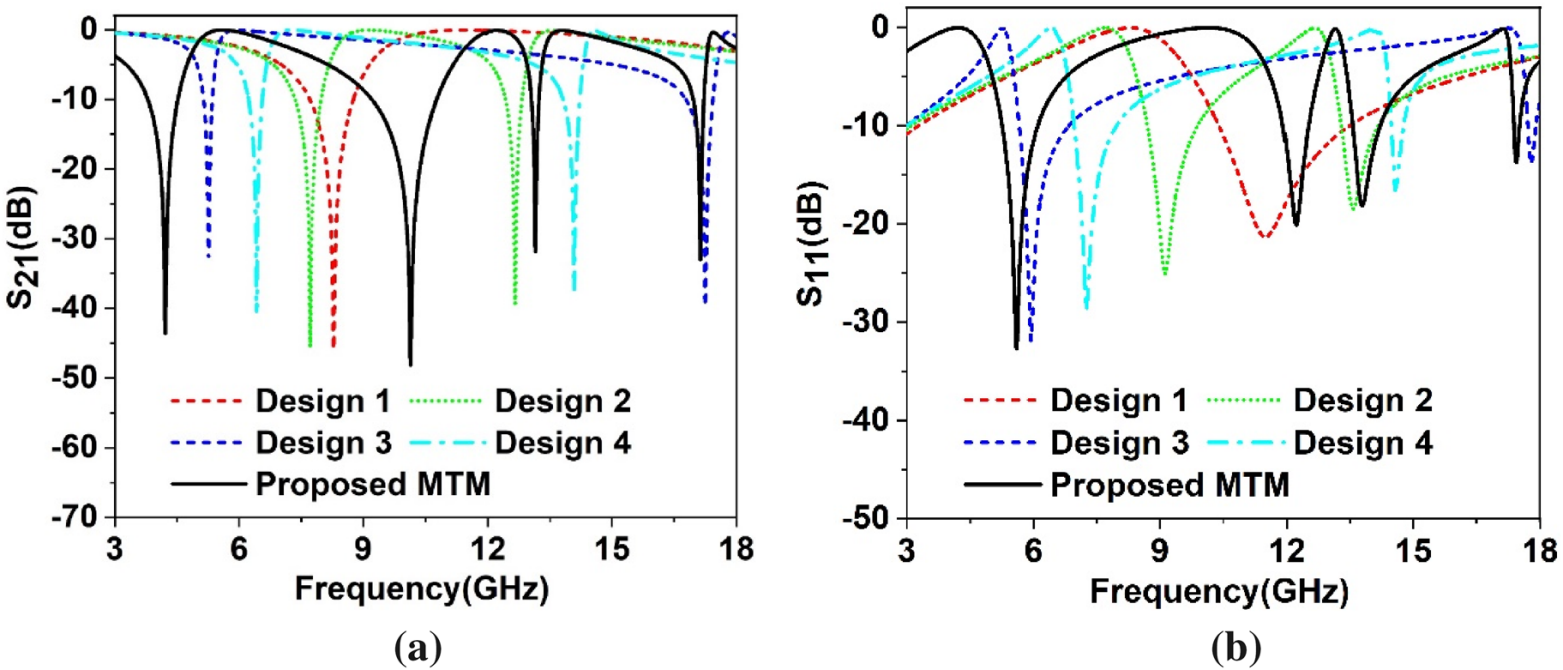
Scattering parameters for evolution steps towards the proposed unit cell: (**a**) Transmission coefficient (S_21_). (**b**) Reflection coefficient (S_11_).

The proposed MTM is finalized through step by step design as shown in Fig. 3 and observing the response of transmission coefficient (S_21_) and reflection coefficient(S_11_) for different design configurations as shown in Fig. 4a,b, respectively. The design is initiated with four split ring resonators of equal dimension at four quadrants of the squared shape substrate. Each ring contains two split gaps: one in horizontal arm and another at vertical arm in such a way that total configurations are axis-symmetric, as shown in Design 1 of Fig. 3. This configuration provides a single resonance of S_21_ at 8.72 GHz followed by the resonance of S_11_ at 11.5 GHz, as shown in Fig. 4a,b, respectively. In design 2, another ring is added inside of the previous ring in each quartile, which contains a split gap at inner vertical arm, as shown in design 2 of Fig. 3. The inclusion of this ring contributes to a new resonance of S_21_ at 12.7 GHz. Moreover, Due to mutual inductance between the two rings, the inductive effect of the first ring modifies, causing a shift of earlier resonance at 7.7 GHz. Two resonances of S_11_ are also happened at 9.13 GHz and 13.58 GHz, as depicted in Fig. 4b. In design 3 of Fig. 3, these two rings of each quartile are interconnected at the point of the split gap of vertical arm of the outer ring, which causes a shift of resonances with first resonance shifts towards lower frequency and second resonance shifts towards high frequency as shown in Fig. 4a,b. This extreme shift of resonances is neutralized by coupling the two rings at bisecting points of top horizontal arms as expressed in design 4 of Fig. 3 and extending the interconnecting metallic strip towards the center of each quartile. The inclusion of these metal strips causes the resonances of S_21_ and S_11_ in between those of Design 2 and Design 3. Now, the proposed MTM is obtained by connecting four quartiles by using a square-shaped metallic strip at the center of the unit cell. Four tuning metallic stubs are extended from this center metal towards two perpendicular axes keeping the total structure symmetric, as shown in Proposed unit cell of Fig. 3. This tuning metal stub helps to obtain four frequency of resonances of S_21_ obtained at 4.2 GHz, 10.14 GHz, 13.15 GHz and 17.1 GHz with corresponding resonances of S_11_ at 5.6 GHz, 12.2 GHz, 13.8 GHz and 17.4 GHz. The information related to S_21_ for various design steps is summarized in Table 2.

**Table 2:** Transmission coefficient (S_21_) of successive steps for the proposed unit cell.

Substructure	Resonance frequency (GHz)	Bandwidth(GHz)	Resonance peak (dB)	Covering bands
Design 1	8.72	1.05	− 48.7	X
Design 2	7.7, 12.7	0.62, 0.4	− 50.9, − 43.9	C, Ku
Design 3	5.26, 17.23	0.21,0.64	− 33.44, − 43.24	C, Ku
Design 4	6.42,14.09	0.33, 0.4	− 42.10, − 33.44, − 14.29	C, Ku
Proposed unit cell	4.20, 10.14, 13.15, 17.1	0.72,1.55,0.17,0.24	− 46.99, − 49.28, − 35.8, − 36.6	C, X, Ku

### Equivalent circuit modelling and simulation

**Figure 5:**
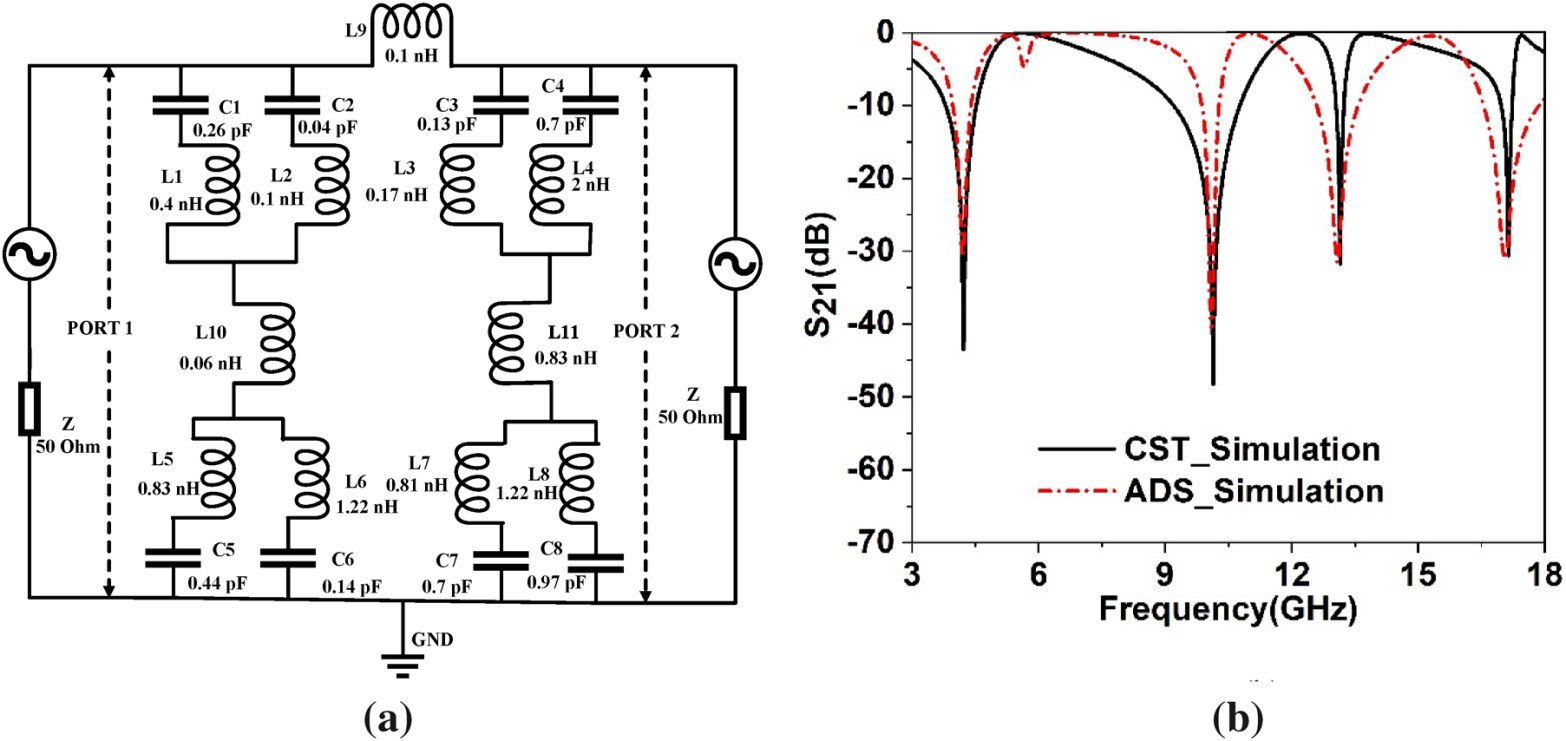
(**a**) Equivalent circuit of the proposed unit cell, (**b**) S_21_ comparison of the equivalent circuit.

The researchers have followed numerous approaches to model the equivalent circuit. In^43^, the cavity model approached has been presented where the resonating element can be considered as an RLC tank circuit, whereas the lumped equivalent circuit approach contemplates the microwave elements consisting of inductance, resistance, capacitance, and conductance^44^. The equivalent circuit of the proposed metamaterial unit cell can be designed by considering the metallic conductor with inductor property since, owing to the current flow, magnetic induction occurs. The split gap of the ring exhibits capacitive effect; thus, every spit ring resonator acts as the resonant tank circuit with inductance L and capacitance C. thus, the split ring acts as a resonator showing resonance at a specified frequency, and it can be controlled by precise control of L and C values with the help of controlling length and thickness of the ring and also controlling the split gap and inter-ring distance. In our present design, each quarter contains two squared split ring resonators interconnected with two metal strips. Each of the split rings can be expressed as series LC circuits. The first quarter of the unit cell can be represented by the two LC circuit pairs *L1*, *C1* & *L2*, *C2*. Similarly, *L3-C3* & *L4-C4*, *L7-C7* & *L8-C8*, *L5-C5* & *L6-C6* are the inductor-capacitor pairs that are represented by other quartiles in the clockwise directions. Since a central metal strip connects four quartiles, this interconnection is expressed by the series inductances *L9*, *L10* and *L11,* where *L10* and *L11* are associated with the two vertical quartiles and *L9* is the interconnecting inductance between two vertical halves. The equivalent circuit of the proposed unit cell is depicted in Fig. 5a. Advanced design system (ADS) is used to confirm whether the equivalent circuit actually presents the proposed unit cell or not in which two ports are connected at two terminals by 50 Ω terminal impedances. The components values are set by tuning the values in ADS so that the S_21_ obtained in ADS circuit simulation matches with the S_21_ obtained in CST. Figure 5b exhibits the S_21_ response in both simulations. As shown in Fig. 5b, both S_21_ are well matched. In the tuning process, component values of *L1-C1* & *L5-C5* pairs are tuned to obtain resonances around 10.14 GHz and 13.15 GHz, respectively. Likely, the amplitude of resonances around these frequencies can be controlled by the inductor-capacitor pairs *L2-C2* and *L6-C6*. In addition, values of *L3-C3* & *L7-C7* show their impacts on the resonances at 17 GHz. The resonance at 4.2 GHz can be obtained by adjusting the values of *L4-C4* & *L8-C8*. Coupling inductance *L9 *can be used to adjust the overall amplitude of S_21,_ whereas *L10* impacts mid-frequency resonances and *L11* exhibits its influence on low and high-frequency resonances.

## Frequency tuning of proposed MTM

**Figure 6:**
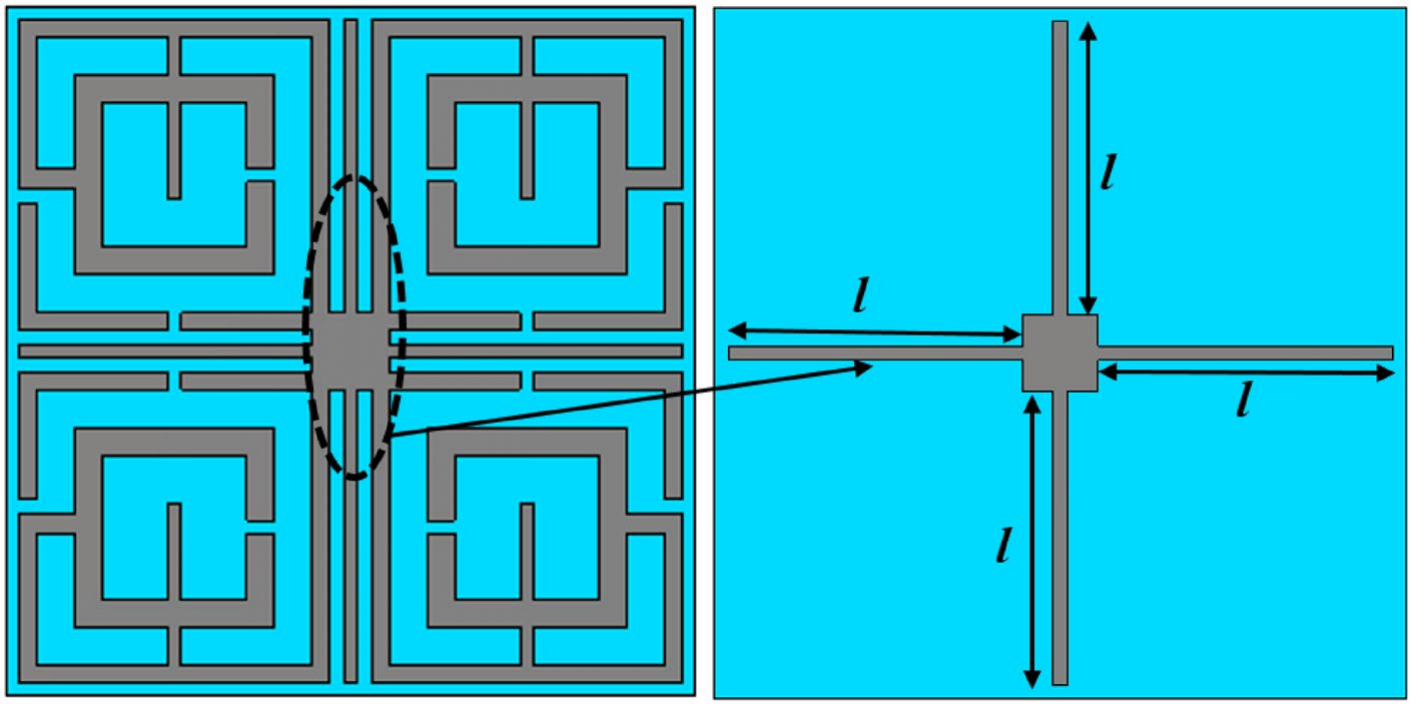
Tuning metal stubs of the proposed MTM unit cell (CST STUDIO SUITE 2019, https://www.3ds.com/products-services/simulia/products/cst-studio-suite)^42^.

**Figure 7:**
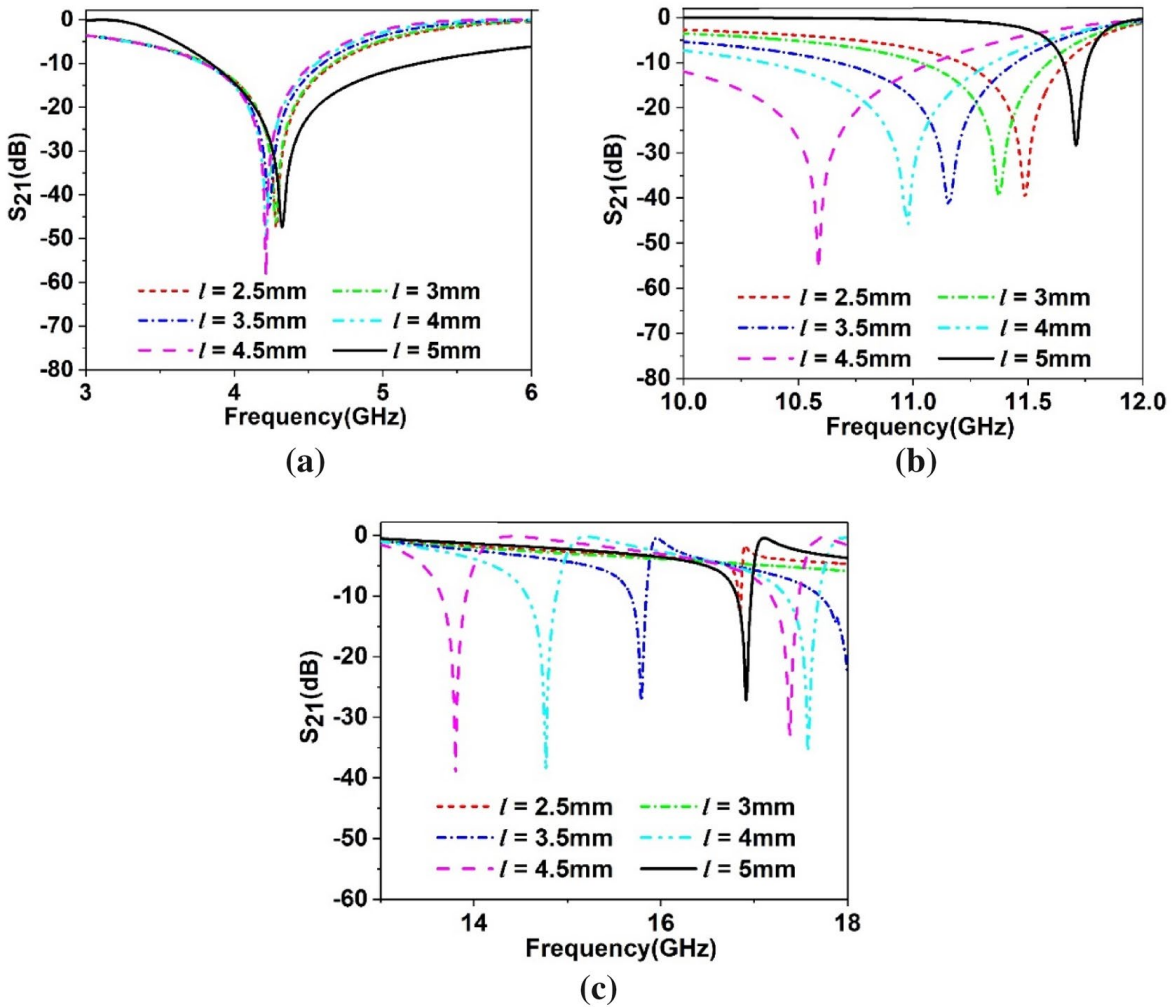
Transmission coefficient (S_21_) for variation of tuning stub length, *l* (**a**) 3 GHz –6 GHz (**b**) 10 GHz – 12 GHz (**c**) 13 GHz – 18 GHz.

The scattering parameters of the proposed MTM can be adjusted as per the application requirement by using four tuning stubs extended horizontal and vertical direction from the center of the structure, as shown in Fig. 6. Length *l* of these stubs are changed simultaneously from maximum length 5 mm to half-length 2.5 mm with an equal distance of 0.5 mm to observe the response of the MTM where *l* is measured from the center of the resonator structure. Resonances of transmission coefficients obtained from these changes of stub length are depicted in Fig. 7a–c. Investigating Fig. 7a, a nominal shift of resonance frequency occurs in the C band in which frequency shifts are within 4.21 GHz – 4.32 GHz for change of *l* with maximum shift is 2.61%. The tuning effect in this band is clearly observable in the case of bandwidth, where it changes within 0.7 GHz – 1.45 GHz with a maximum percentage bandwidth change of 107%. In X band, shift of resonance frequencies are more pronounced where a nearly linear shift in resonance frequencies is observed within ranges from 10.58 GHz to 11.5 GHz, as shown in Fig. 7b. The maximum bandwidth of 1.2 GHz with a center frequency of 10.58 GHz is exhibited in this band, whereas the minimum bandwidth is 0.11 GHz with resonance at 11.7 GHz. In Fig. 7c, responses of the MTM in the X band are depicted in which dual resonances are observed for *l* = 4 mm and 4.5 mm. In this band, the resonances are spread over a large frequency region extended from 13.8 GHz to 17.4 GHz with a minimum bandwidth of 0.16 GHz and a maximum of 0.46 GHz with resonances at 16.85 GHz and 16.9 GHz, respectively for *l* = 2.5 mm and 5 mm, respectively. Noteworthy to mention that maximum length, *l* = 5 mm, contributes to maximum bandwidth at C band resonance and minimum bandwidth of X and Ku band resonances. At this length, horizontal metal stubs are adjacent to the PEC boundary, whereas vertical stubs are closest to the PMC boundaries. S_21_ response of the MTM for various lengths of the tuning stub is summarized in Table 3.

**Table 3:** Performance comparison of the MTM for different stub lengths in terms of resonance frequency and bandwidth.

Length (mm)	Resonance frequency (GHz)	Bandwidth (GHz)	Length (mm)	Resonance frequency (GHz)	Bandwidth (GHz)
2.5	4.28, 11.5, 16.85	0.83, 0.46, 0.04	3	4.28, 11.37	0.8, 0.57
3.5	4.23, 11.15, 15.8	0.8, 0.77, 0.16	4	4.21, 10.98, 14.8, 17.6	0.75, 0.96, 0.3, 0.37
4.5	4.21, 10.58, 13.8, 17.4	0.7, 1.2, 0.2, 0.3	5	4.32, 11.7, 16.9	1.45, 0.11, 0.16

**Figure 8:**
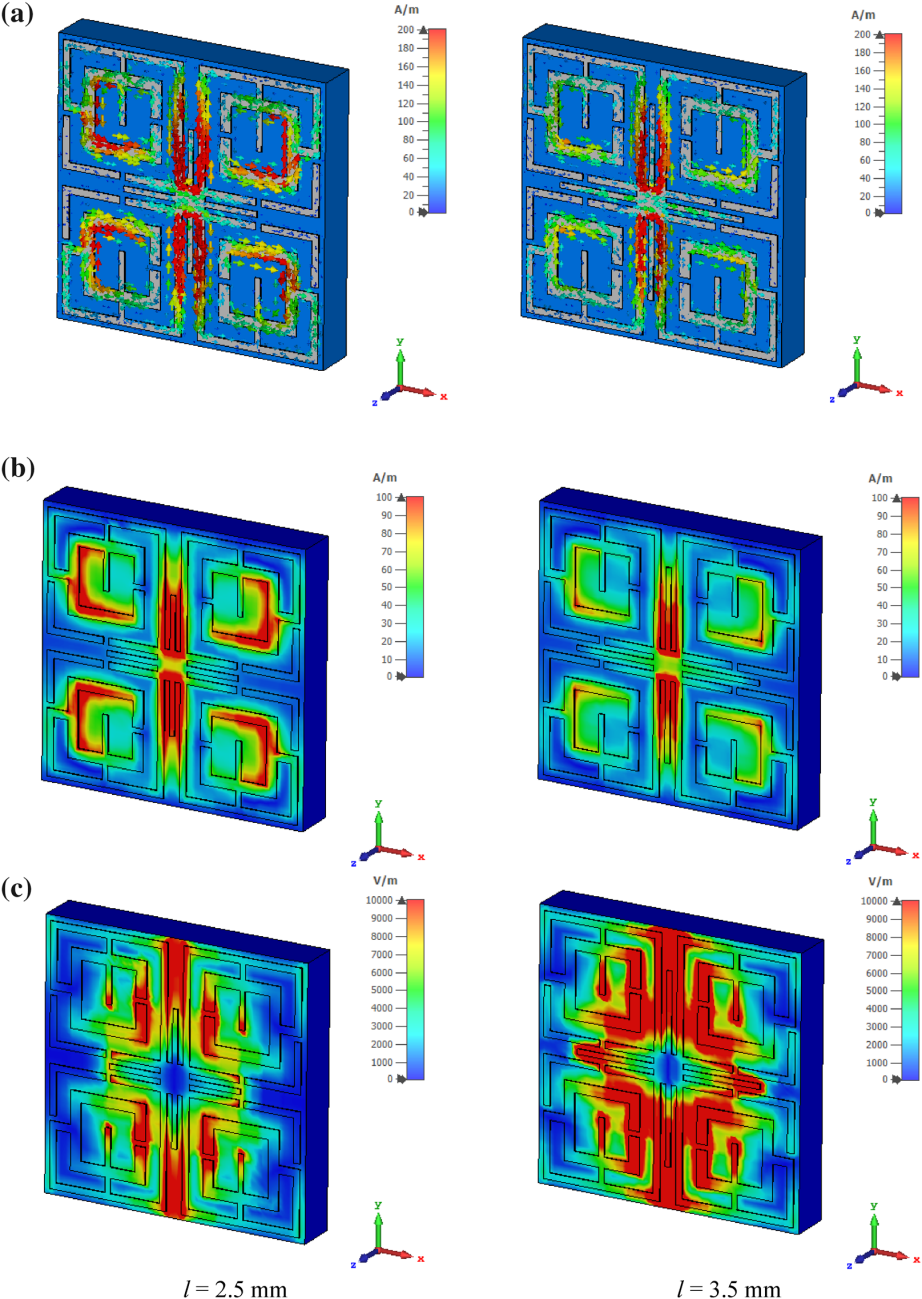
Analysis of EM wave interaction at 11.5 GHz for different length of tuning metallic stubs: (**a**) surface current (**b**) magnetic field (**c**) electric field (CST STUDIO SUITE 2019, https://www.3ds.com/products-services/simulia/products/cst-studio-suite)^42^.

The electromagnetic behavior has been investigated to understand the frequency tuning phenomena for different lengths of tuning stubs by analyzing electric and magnetic fields at a particular frequency. Figure 8 shows surface current, electric field and magnetic field distribution of the MTM at 11.5 GHz for two different lengths of tuning stubs, *l* of 2.5 mm and 3.5 mm. At 11.5 GHz, the resonance of S_21_ occurs when the tuning stub length is 2.5 mm. Surface current density parameters reported in Fig. 8a are obtained by applying an external excitation wave. Plain wave with linear excitation is applied by using two waveguide ports which provides the opportunity to simulate an incident wave located a large distance from the observed object. Open boundary condition is defined in the direction of the incidence and input signal contains pulse in Gaussian shape. Figure 8a shows that inner rings in the upper two quartiles provide routes to flow current in anticlockwise direction , whereas currents in the same two rings of other two quartiles flow in clockwise direction. Thus, these two currents induce magnetic fields that are of opposite polarities. Hence, magnetic dipoles are created between two vertical quartiles. Similarly, in the upper half of the patch, a strong anticlockwise current loop is formed through the two vertical arms of the outer rings near the metallic tuning stub that produces a strong magnetic field. Likely, a strong opposite magnetic field exists in the lower half due to clockwise current flow through similar arms. Thus, strong magnetic dipoles are created that alter the resonator's inductance, resulting in resonance at this frequency. The magnetic field distribution is presented in Fig. 8b. Electric field distribution in Fig. 8c expresses that a strong electric field exists at splits gaps of four inner rings that contribute to the variation of capacitance. A comparison of surface current distribution presented in Fig. 8a reveals that as *l* increases to 3.5 mm, the current density in each ring decreases significantly, resulting in a weak magnetic field (shown in Fig. 8b). Due to the lack of a strong magnetic dipole, no resonance occurs at this frequency. In Fig. 8c, a strong electric field is observed for *l* = 3.5 mm compared to *l* = 2.5 mm, but this field is distributed and random, and no strong electric dipole is constituted. Thus, *l* = 3.5 mm lacks supporting a resonance of S_21_ at this frequency of observation. It can be concluded that changing the length of the tuning metallic stub changes the orientation of the induced electromagnetic field contributing to modification of inductance and capacitance associated with the rings and eventually changes the frequency of resonances.

**Figure 9:**
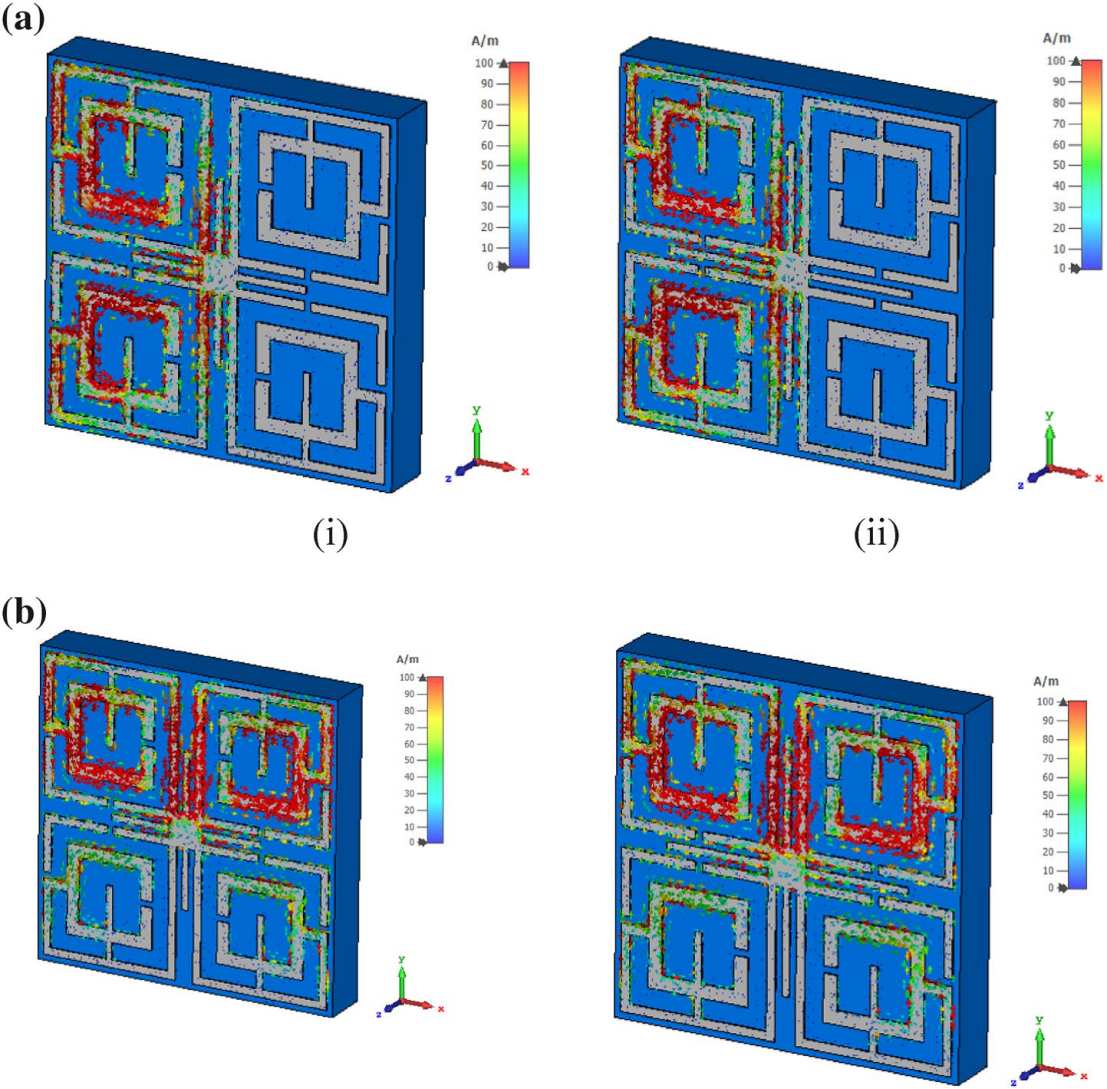
(**a**) Surface current distribution at 11.5 GHz for the wave guide port oriented in X direction. (i) for *l* = 2.5 mm, (ii) for *l* = 3.5 mm. (**b**) Surface current distribution at 11.5 GHz for the wave guide port oriented in Y direction. (i) for *l* = 2.5 mm, (ii) for *l* = 3.5 mm (CST STUDIO SUITE 2019, https://www.3ds.com/products-services/simulia/products/cst-studio-suite)^42^.

The surface current distribution in the MTM for two different length of tuning stub is further analyzed for two different orientation of the wave guide ports in X and Y directions. Figure 9a shows current distribution for incident wave directional to X-axis. From Fig. 9a it is observed that more induced current is noticed near the source port and thus current concentration is higher in the left vertical half of the unit cell. A nominal increment of current is noticed near the tuning stub when its length is increased from 2.5 mm to 3.5 mm due induced effect among the metallic lines. Current distribution pattern presented in Fig. 9b shows that current is more concentrated in the upper half of the unit cell as the source port is radiated in the direction of negative Y-axis. Increasing the tuning stub length helps to increase the current density through it. A comparison of current distribution pattern presented in Fig. 8a shows that when the ports are oriented in the Z axis current is symmetrically distributed among the four quartiles of the unit cell. This distribution is due to fact that incident electromagnetic wave is exposed on the resonator in such a way that every quartile of the resonator interact with the incident wave symmetrically. On the other hand, for X and Y direction orientation of the source ports impose the electromagnetic wave from sideways thus nearest quartiles interacts with the incident wave more compared to the far end quartiles.

## Result and analysis

In this section, the effective parameters are of the proposed MTM unit cell have been extracted using the post-processing module of CST microwave studio that uses robust retrieval method^45^ with the knowledge of S_21_ and S_11_ to extract the parameters, and then the obtained result is analyzed. The power associated with the MTM during simulation is also investigated. The behaviors of the electric field, magnetic field and surface current for different resonances are also studied. Experiments on transmission coefficient have been discussed, and the result is analyzed in comparison with the simulated result. A comparison of the proposed MTM with some recent works is accomplished in this part.

### Analysis of effective parameters

**Figure 10:**
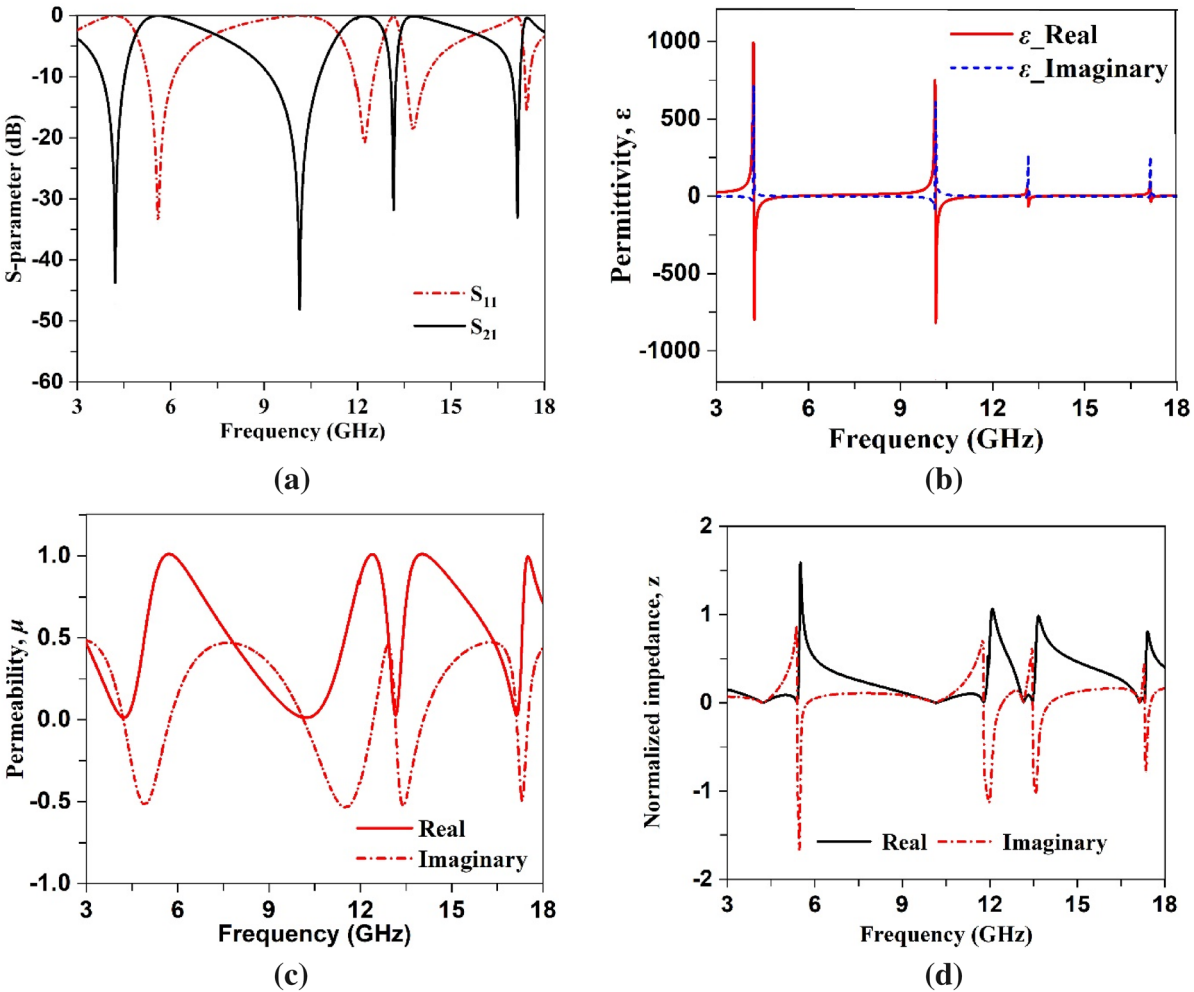
(**a**) Scattering parameters plot (**b**) permittivity (**c**) and permeability plot (**d**) normalized impedance plot.

The transmission and reflection coefficient, permittivity, permeability and normalized impedance obtained from the CST microwave studio are presented in Fig. 10a–d. As shown in Fig. 10a, four resonances of S_21_ are followed by corresponding resonances of S_11,_ thus indicating electrical resonance. The permittivity plots exhibit resonances at frequencies of 4.2 GHz, 101.4 GHz, 13.15 GHz and 17.1 GHz as shown in Fig. 10b, whereas permeability is near zero at the resonance frequencies as shown in Fig. 10c. The frequency ranges of negative permittivity and near zero permeability are summarized in Table 3. Permittivity is also related to the plasma frequency as^46^:

3 $$\varepsilon_{ } = 1 - \frac{{\omega_{p}^{2} }}{{\omega^{2} }}$$

where $\omega_{p}^{ }$ and $\omega$ are the plasma frequency and frequency of the electromagnetic wave, respectively. Equation (3) indicates that when $\omega < \omega_{p}^{ }$ permittivity will be negative and $\omega = \omega_{p}^{ }$ makes the effective permittivity zero. Figure 10b shows that negative permittivity approaches to zero at 5.47 GHz, 11.89 GHz, 13.65 GHz and 17.6 GHz are related to the plasma frequencies of the proposed MTM. Moreover, permeability can also be presented as^41^:

4 $$\mu = 1 - \frac{{F\omega^{2} }}{{\omega^{2} - \omega_{0}^{2} + i\omega \Gamma }}.$$

In this equation, $F$, $\omega_{0}^{ }$ and $\Gamma$ are the fractional area of the patch, resonance frequency and dissipation factors, respectively where $\omega_{0}^{ }$ depends on the inductance and capacitance associated with the patch. Investigating Eq. (4), it is concluded that permeability depends on the area of the resonating patch and operating frequency and when wave frequency approaches the frequency of resonance, permeability decreases from unity. Permeability plot depicted in Fig. 10c provides permeability of 0.01, 0.01, 0.03 and 0.02 at 4.26 GHz, 10.14 GHz, 13.14 GHz, 17.1 GHz respectively. With an investigation of Maxwell curl equations, $\nabla \times E = i\omega \mu H$ and $\nabla \times H = - i\omega \varepsilon E$ indicates that with $\varepsilon \approx 0$ or $\mu \approx 0$ decoupling occurs in the electric and magnetic field. In case of time varying electromagnetic wave electric and magnetic field are coupled with each other as per the mentioned two Maxwell’s curl equations. The first one resembles the Faraday’s law of electromagnetic coupling indicating that varying magnetic field induced an electric field. One the other hand, second equation represents the amperes law indicating the generation of magnetic field due to the electric field. Thus, electric and magnetic fields are associated with each other and electrometric waves propagates through space due to this electromagnetic coupling. But, as per the above mentioned equation, when $\mu \approx 0$ , curl of the electric field approaches to zero. Similarly, for $\varepsilon \approx 0$ magnetic field becomes zero. Thus, electromagnetic field decoupling occurs in case of near zero permeability and permittivity. The normalized impedance of the proposed MTM is presented in Fig. 10d. The impedance, z of the proposed design, contains real and imaginary parts satisfying the relation, $z = R + jX$. This impedance is correlated with the reflection coefficient (S_11_) and expressed as^47^:

5 $$S_{11} = \frac{{z - z_{0} }}{{z + z_{0} }},\;{\text{where}}\;{\text{free}}\;{\text{space}}\;{\text{impedance}}\;z_{0} = \sqrt {\frac{{\mu_{0} }}{{\varepsilon_{0} }}} = {377}\Omega .$$

A closer look at the normalized impedance reveals that real and imaginary parts of the normalized impedance are positive in the vicinity of negative permittivity, indicating that proposed MTM acts as a passive media in these frequency ranges^48^.

**Figure 11:**
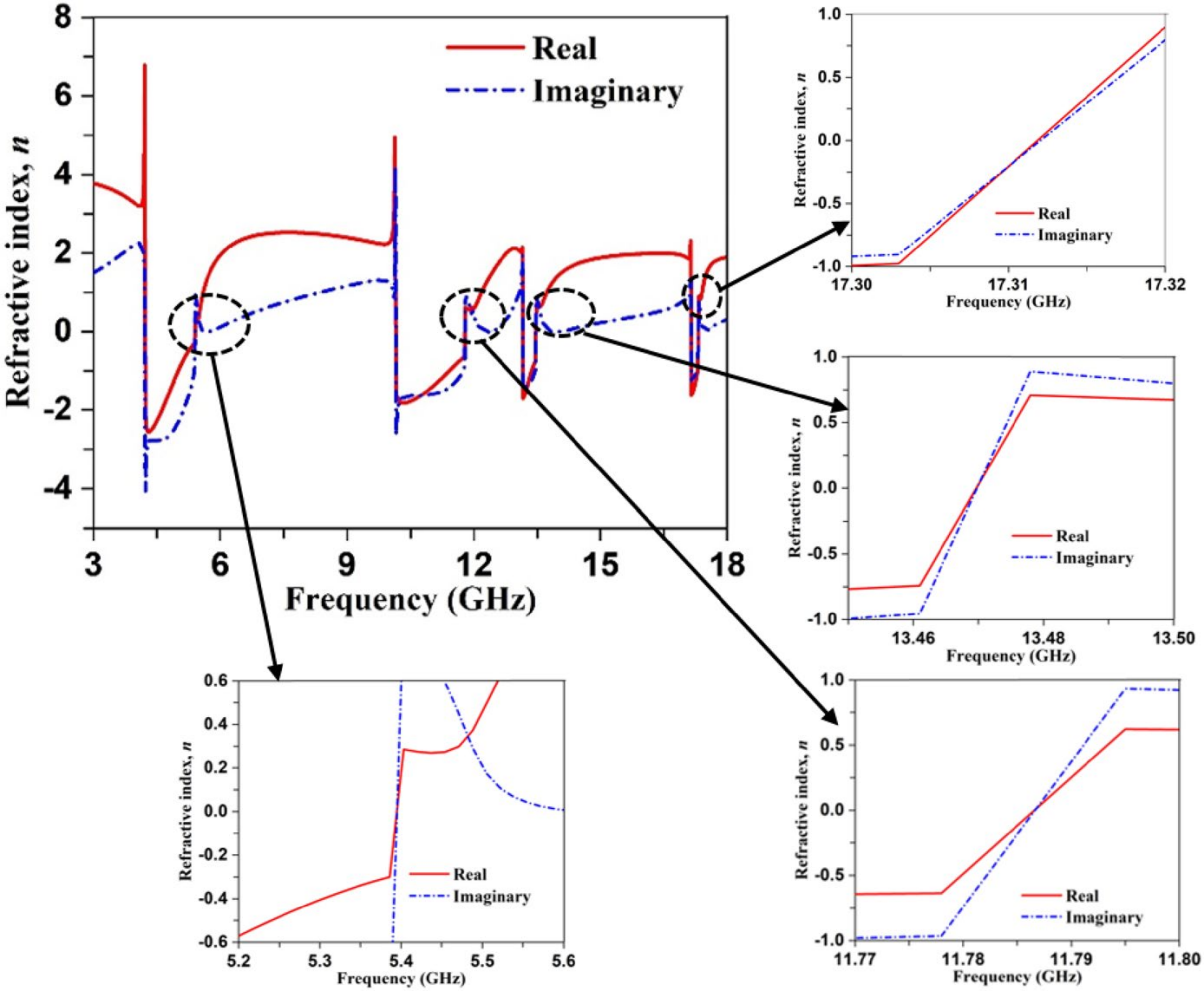
Refractive index of the proposed MTM unit cell.

The refractive index depicted in Fig. 11 shows that it undergoes positive to negative transition at the resonance frequencies. Table 4 contains the range of frequencies where the refractive index shows the negative values. Since refractive index,$n = \sqrt {\mu \varepsilon }$. Correlating with the Eq. (3), it can be concluded that when electromagnetic wave frequency increases towards the plasma frequency, the refractive index approaches zero. Near-zero refractive indices are observed in the frequency ranges from 5.2 GHz –5.5 GHz, 11.7 GHz –11.8 GHz, 13.45 GHz –13.5 GHz, and 17.31 GHz–17.32 GHz in Fig. 11 with zero real and imaginary values of refractive index at 5.37, 11.78, 13.47 and 17.315 GHz. This near-zero refractive index is realized due to permittivity closer to zero. A comparison between Figs. 10a and 11 reveals that zero refractive indexes are obtained in the stop band of transmission coefficients.

**Table 4:** Extracted data for the proposed unit cell.

Parameter	Frequency range (GHz)	Extracted property
Permittivity, ϵ	4.22–5.47, 10.14–11.89, 13.15–13.65, 17.14–17.36	ϵ < 0
Permeability, μ	3.89–4.50, 9.37–10.80, 13.03–13.30, 16.97–17.26	μ ~ 0
Refractive index, n	4.23–5.37, 10.17–11.80, 13.17–13.51, 17.09–17.31	*n* < 0
Normalized impedance, z	4.23–5.386, 9.22–11.84, 13.08–13.51, 17.01–17.33	z ~ 0

### Power analysis

**Figure 12:**
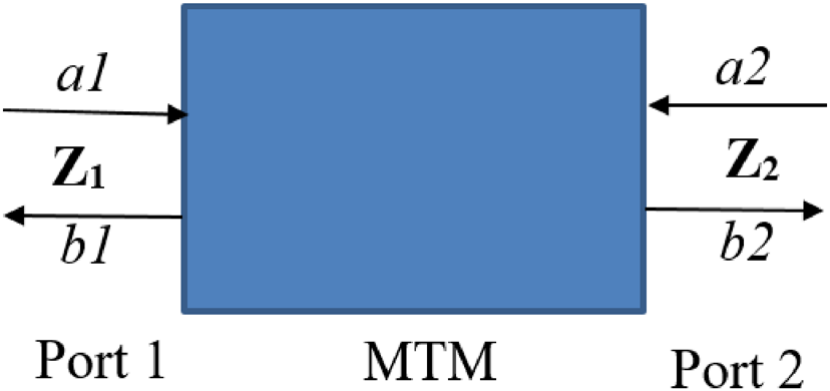
Two port system representation of proposed MTM.

The power associated with MTM has been observed through simulations with the connection of two ports. To analysis the power, the MTM with two ports can be represented by the block diagram shown in Fig. 12, where *a*_*1*_, *a*_*2*_ are incoming power, *b*_*1*_, *b*_*2*_ are outgoing power, Z_1_, Z_2_ are impedances associated with port 1 and 2. The interrelation of these waves with the S-parameter can be presented as:

6 $$\left[ {\begin{array}{*{20}c} {b_{1} } \\ {b_{2} } \\ \end{array} } \right] = \left[ {\begin{array}{*{20}c} {S_{11} S_{12} } \\ {S_{21} S_{22} } \\ \end{array} } \right]\left[ {\begin{array}{*{20}c} {a_{1} } \\ {b_{1} } \\ \end{array} } \right],\;{\text{where}}\;a_{i} = \frac{1}{2}\left( {\frac{{V_{i} }}{{\sqrt {Z_{i} } }} + \sqrt {Z_{i} } I_{i} } \right),\;b_{i} = \frac{1}{2}\left( {\frac{{V_{i} }}{{\sqrt {Z_{i} } }} - \sqrt {Z_{i} } I_{i} } \right)\;{\text{for}}\;i = {1},{2}{\text{.}}$$

The overall power by which the MTM is excited by two ports can be expressed as:

7 $$P_{stimulated} = \frac{1}{2}\mathop \sum \limits_{n = 1}^{2} A_{n}^{2} ,\;{\text{where}}\;A\;{\text{is}}\;{\text{the}}\;{\text{amplitude}}\;{\text{of}}\;{\text{wave}}\;{\text{of}}\;{\text{the}}\;{\text{signal}}\;{\text{generator}}.$$

8 $${\text{Total}}\;{\text{power}}\;{\text{accepted}}\;{\text{by}}\;{\text{any}}\;{\text{port}}\;{\text{is}}\;{\text{expressed}}\;{\text{as}},\;P_{i} = a_{i}^{2} - b_{i}^{2} .$$

**Figure 13:**
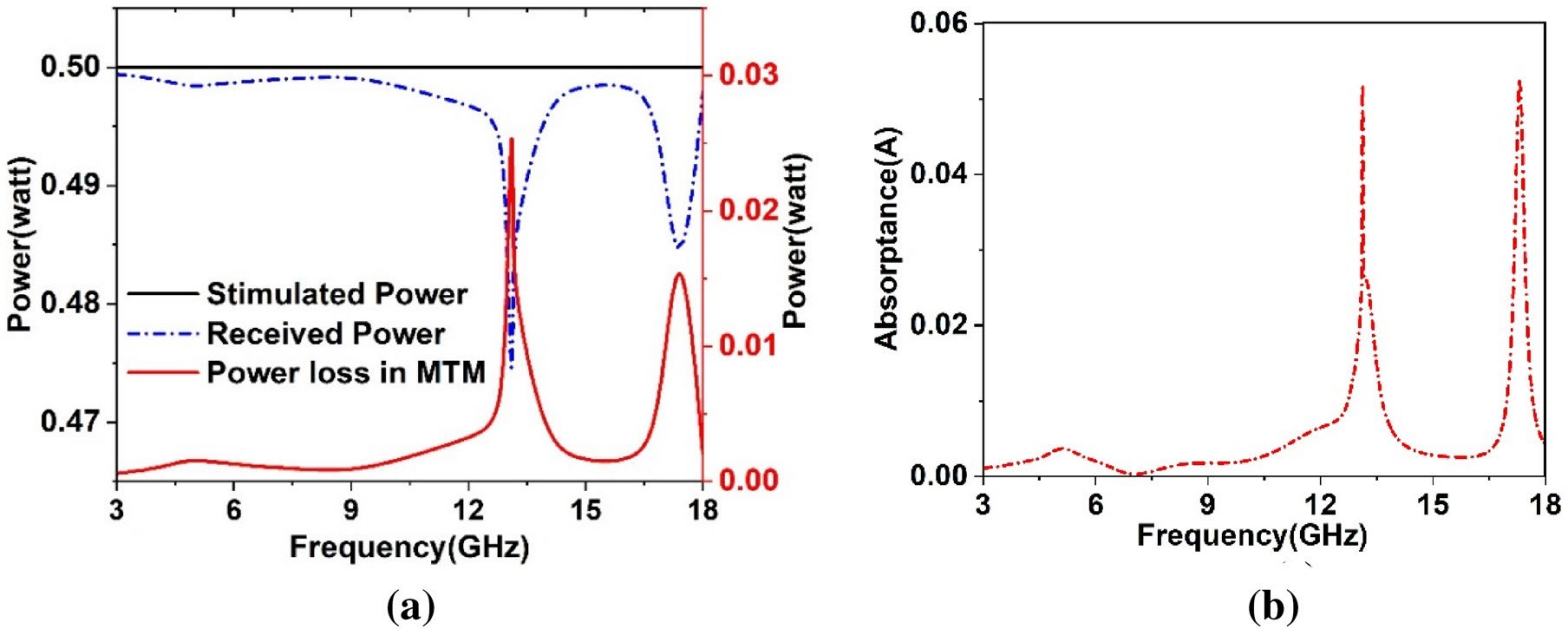
(**a**) Two port system of proposed MTM. (**b**) Power plots for excitation, loss and received at ports.

Figure 13a shows the power graphs associated with MTM, indicating that the proposed MTM unit cell accepted a very small amount of power absorbed or radiated by the structure. The loss is almost zero with two spikes around 13.2 GHz and 17.3 GHz which is within 3–5% of the total stimulated power. Thus, the proposed MTM exhibits a low loss passive medium for microwave applications. The absorption that occurred in the MTM is investigated to search the origin of the power loss. When electromagnetic wave is exposed to the resonating structure of the MTM, a portion of the incident wave is reflected, some amount is absorbed, and the rest is transmitted through the substrate. The amount of absorbed energy relating to the S-parameters can be expressed as:

9 $${\text{Absorptance}},\; A = 1 - S_{11}^{2} - S_{21}^{2} .$$

The absorbed power of the proposed MTM is presented in Fig. 13b, which shows a very low level of absorption, having nearly zero all over the frequency ranges of interest with an exception around 13.2 GHz and 17.3 GHz. The absorption peaks around these two frequencies are approximately 5%. Though power absorptions in these two frequencies are nearly the same but loss at the 13.2 GHz band is high compared to the 17.3 GHz band, it is due to the more radiation loss at 13.2 GHz, which is accumulated with the absorption loss, thus provides a little higher loss compared to 17.3 GHz.

### Effect of port relocation

**Figure 14:**
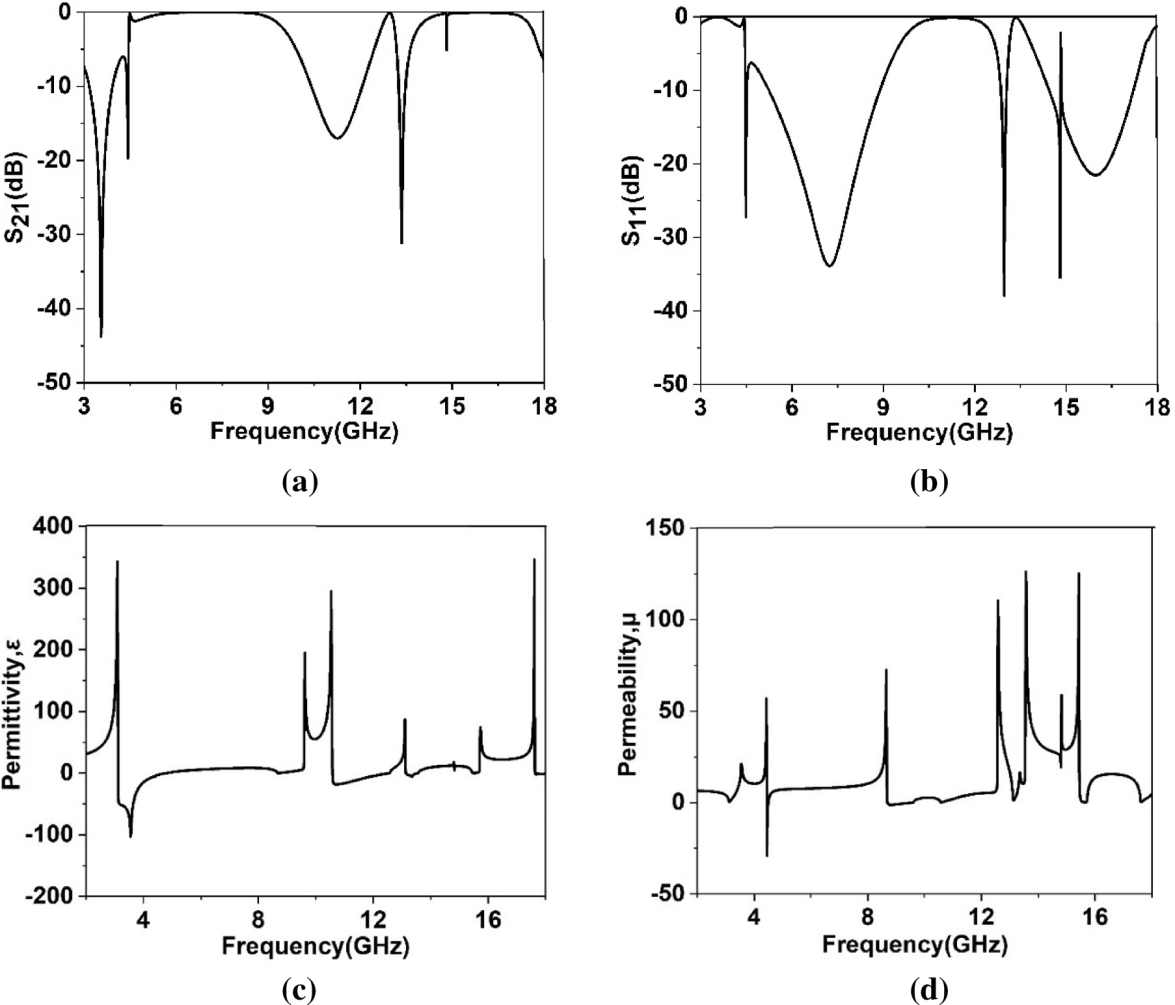
(**a**) Transmisson coefficient (S_21_) (**b**) reflection coefficien (S_11_) (**c**) permittivity (**d**) permeability where ports are assigned in X axis.

**Figure 15:**
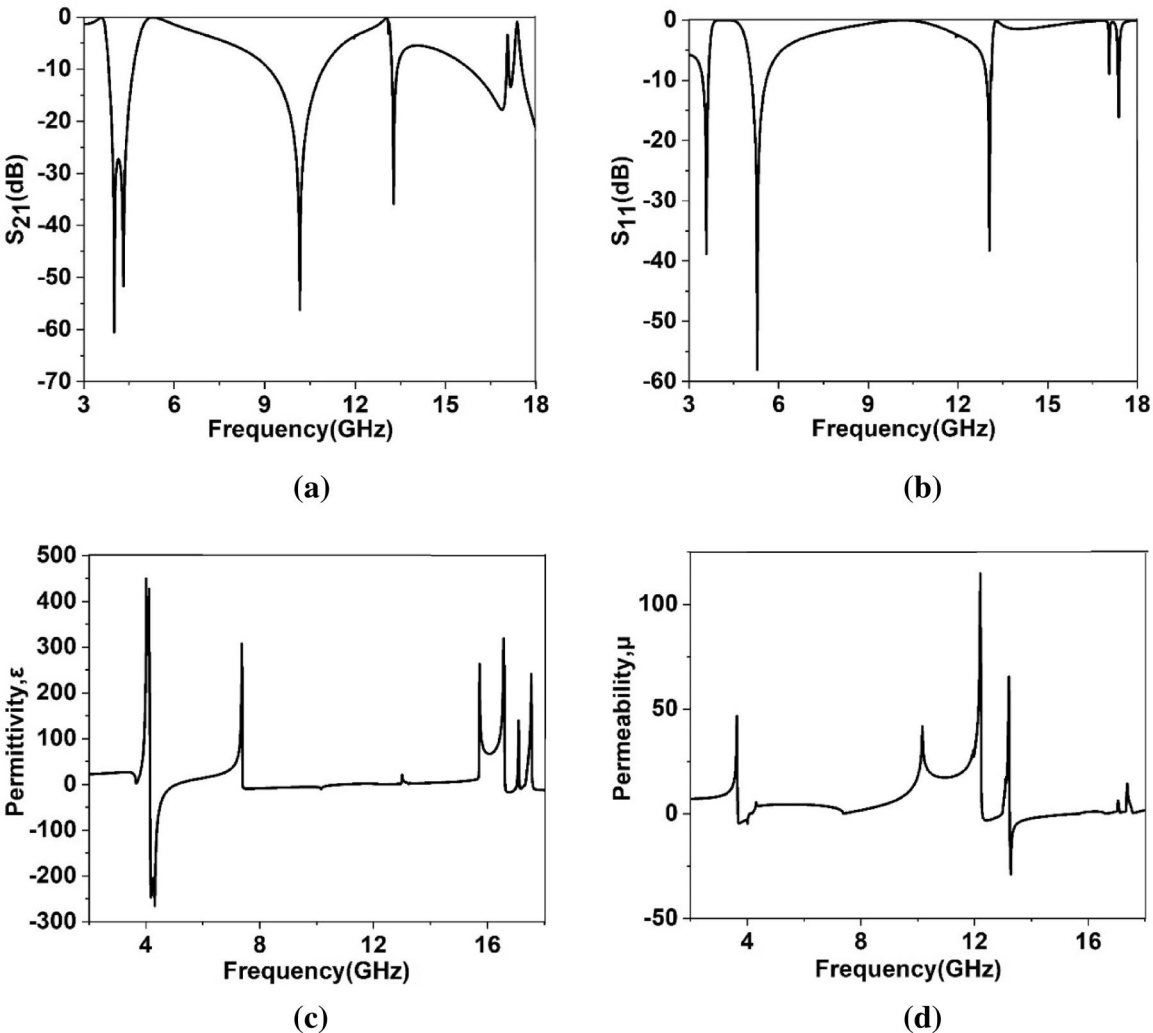
(**a**) Transmisson coefficient (S_21_) (**b**) reflection coefficien (S_11_) (**c**) permittivity (**d**) permeability where ports are assigned in Y axis.

The previous results obtained for the port oriented in the Z axis. It is also necessary to study the effect of different positions of the ports on the scattering and effective parameters by relocating them in X and Y directions. In Fig. 14a–d, transmission coefficient, reflection coefficient, permittivity and permeability plots are exhibited when ports are assigned in X axis. As shown in Fig. 14a, transmission spectra exhibits four resonances at 3.55 GHz, 4.4 GHz, 11.25 GHz and 13.34 GHz with magnitudes of − 43.8 dB, − 19.7 dB, − 17 dB and − 31 dB, respectively covering S, C, X and Ku bands. On the other hand, reflection spectra shows the resonances at 4.48 GHz, 7.25 GHz, 12.97 GHz, 14.8 GHz and 15.98 GHz as expressed in Fig. 14b. The permittivity graph presented in Fig. 14c shows negative values extended form 3.12 GHz – 4.8 GHz, 10.58 GHz – 12.55 GHz, 13.14 GHz – 13.57 GHz. Additionally, negative value of the permeability is obtained in the frequency ranges from 4.45 GHz – 4.5 GHz and 8.69 GHz – 9.58 GHz as depicted in Fig. 14d. Thus, in this orientation of the waveguide port double negative property is obtained extending from 4.45 GHz to 4.5 GHz. The effect of the port relocation in Y direction is described using the Fig. 15a–d. S_21_ response presented in Fig. 15a exhibits resonances at 4 GHz, 4.3 GHz, 10.16 GHz, 13.3 GHz and 16.86 GHz, whereas S_11_ response shows the resonances at 3.58 GHz, 5.28 GHz, 13 GHz and 17.37 GHz as depicted in Fig. 15b. As depicted in Fig. 15c, in this orientation of excitation field, negative permittivity is obtained in frequency ranges 4.16 GHz – 5 GHz, 7.4 GHz – 10.74 GHz, 16.6 GHz – 17.1 GHz, 17.2 GHz –17.3 GHz, whereas permeability graph exhibits negative values within the frequency ranges 3.67 GHz – 4.1 GHz, 12.27 GHz – 12.97 GHz, 13.2 GHz – 15.6 GHz (as shown in Fig. 15d). Thus, The MTM unit cell exhibits single negative behaviors when ports are oriented in Y directions.

### Electric field, magnetic field and surface current analysis

**Figure 16:**
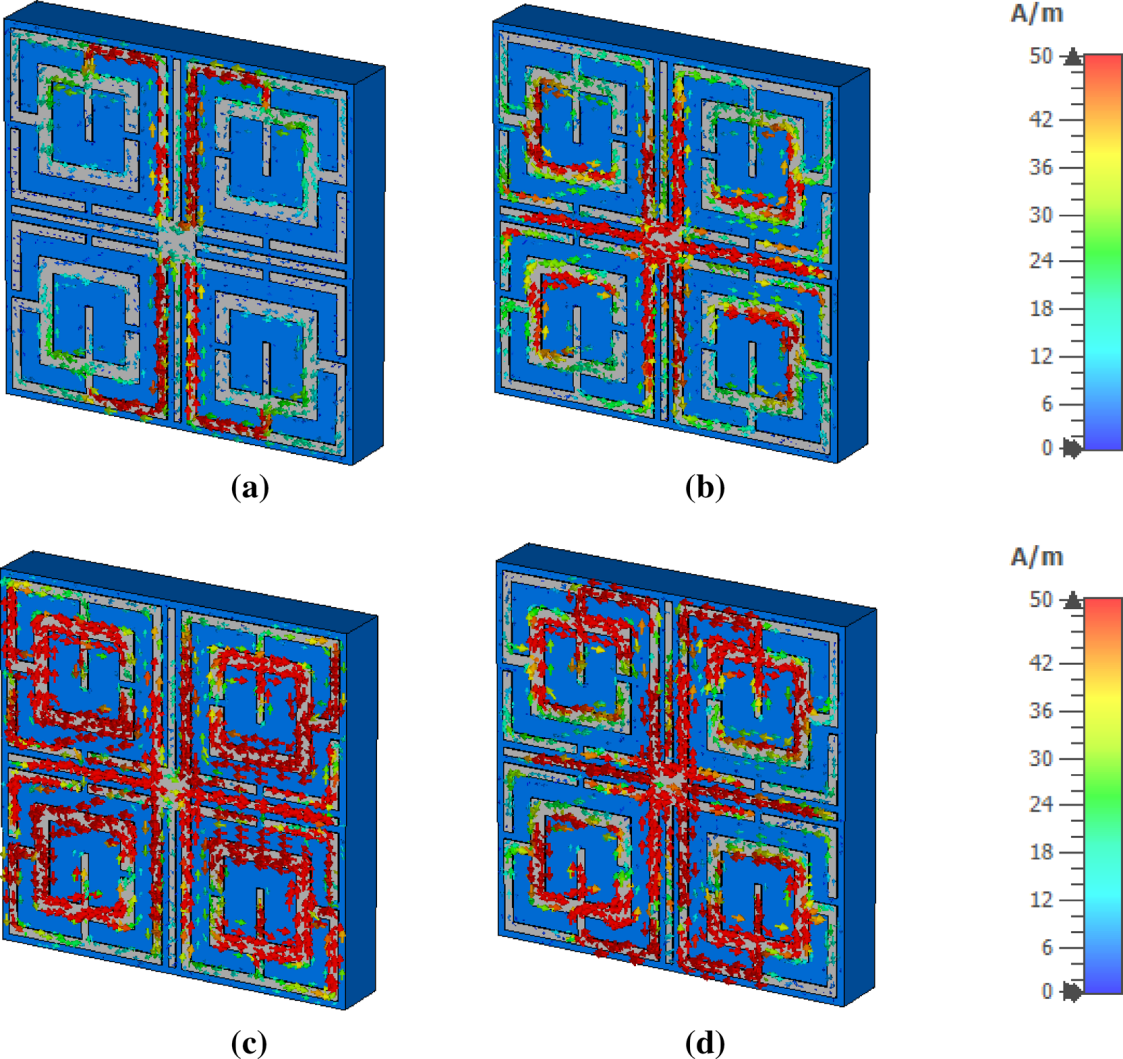
Surface current distribution: (**a**) 4.2 GHz, (**b**) 10.14 GHz, (**c**) 13.15 GHz, and (**d**) 17.1 GHz.

**Figure 17:**
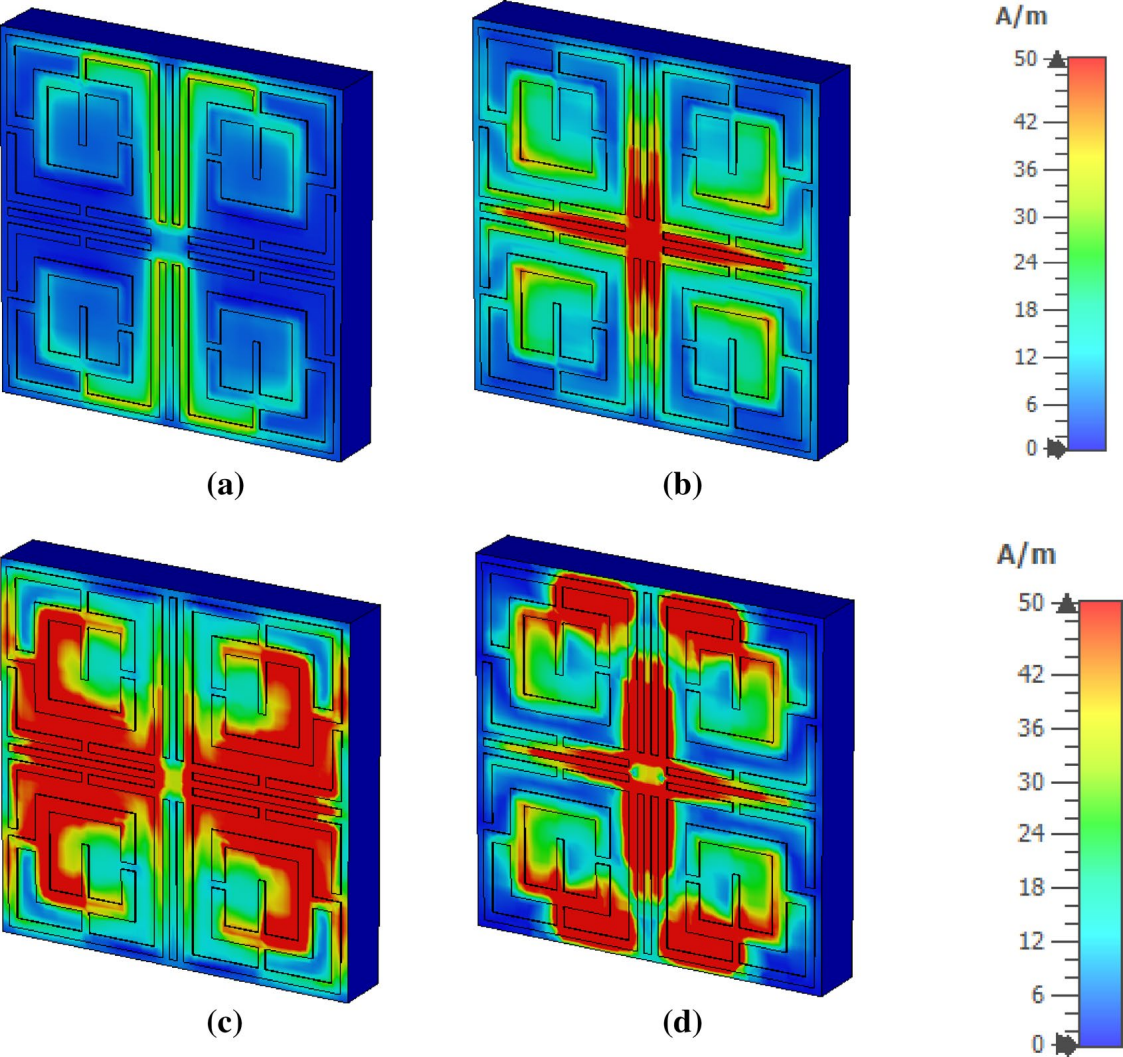
Magnetic field distribution (**a**) 4.2 GHz, (**b**) 10.14 GHz, (**c**) 13.15 GHz, and (**d**) 17.1 GHz.

**Figure 18:**
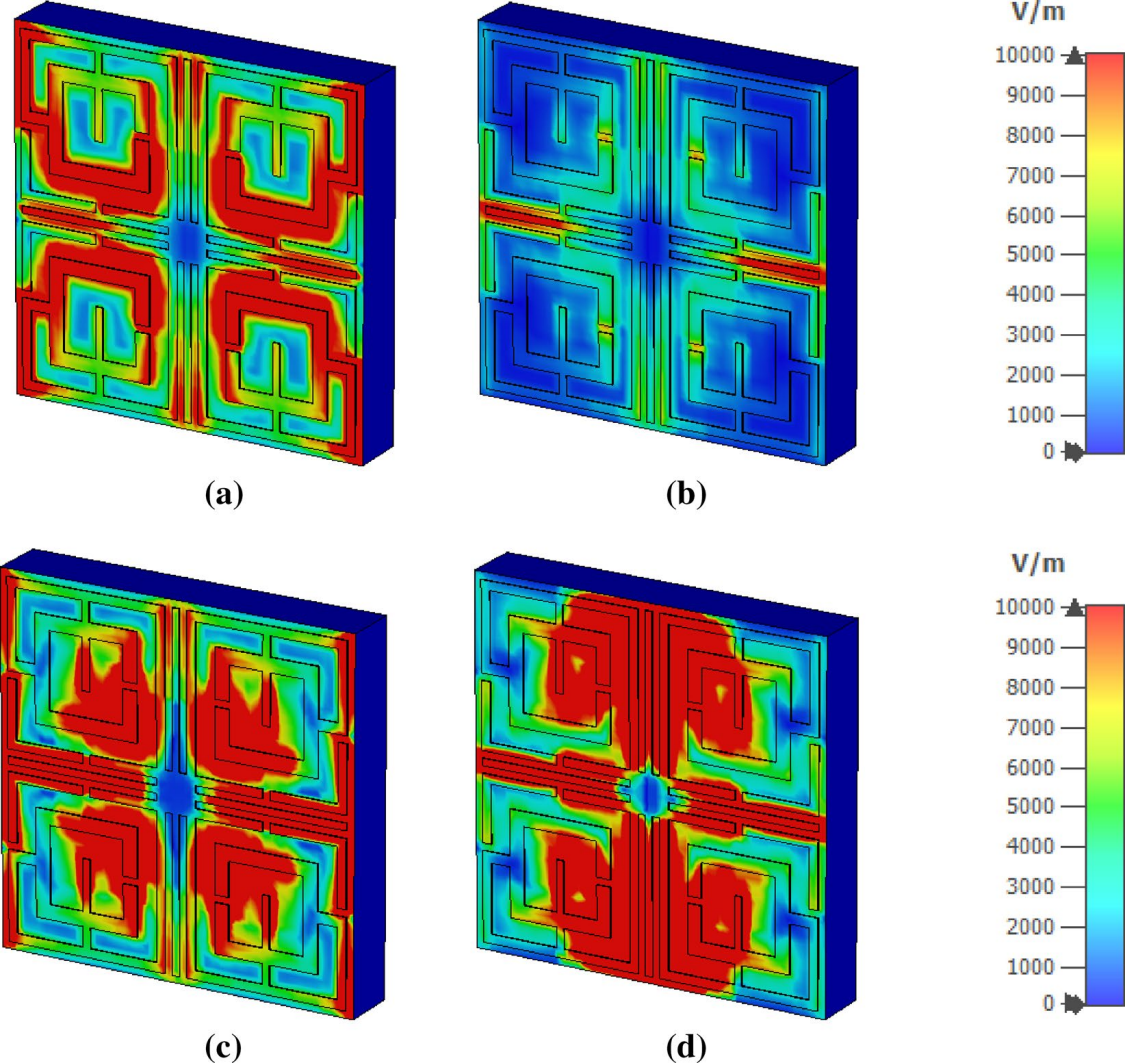
Electric field distribution: (**a**) 4.2 GHz, (**b**) 10.14 GHz, (**c**) 13.15 GHz, and (**d**) 17.1 GHz (CST STUDIO SUITE 2019, https://www.3ds.com/products-services/simulia/products/cst-studio-suite)^42^.

The metamaterial properties can be well understood with the help of surface current, electric and magnetic field analysis. The interrelation between these three quantities along with the material is presented in Maxwell’s equations^40^. In Fig. 16a, surface current at the resonance frequency, 4.2 GHz, mainly flows through the vertical arms of the outer rings, and two current loops form in the upper and lower halves. These oppositely flowing currents give rise to the magnetic field near these portions, as shown in Fig. 17a. Strong electric fields are observed through other parts of the resonator at this frequency as depicted in Fig. 18a. At 10.14 GHz, additional current flows through the horizontal metallic stubs and inner rings, as shown in Fig. 16b. An increased current is also noticed through vertical tuning stubs. This strong current creates strong magnetic dipoles between the upper and lower halves of the resonator, as shown in Fig. 17b. A reduced electric field is noticed all over the structure except near the edges of the horizontal and vertical tuning stubs at this frequency of resonance, as shown in Fig. 18b. At frequency 13.15 GHz, high intensity current is observed all over the resonating part, especially at the middle of the resonator, as shown in Fig. 16c, which generates a high magnetic field in this region as depicted in Fig. 17c. The strength of the electric field is high in other parts of the resonator, as shown in Fig. 18c. Finally, at 17.1 GHz, the current orientation is shifted a little bit with as depicted in Fig. 16d. Horizontal arms of the outer rings contribute to a significant amount of current. The amount of current through the inner ring is reduced significantly. Corresponding magnetic and electric field distribution is shown in Figs. 17d and 18d, respectively. The magnetic field is concentrated at the middle of the structure surrounding the tuning metal stubs and around the interconnection of the two rings. On the other hand, a high intensity electric field is widespread near the vertical tuning stubs extending toward consecutive arms of the two rings. Electric field distribution is also high around horizontal metallic stubs concentrated in a narrow area. Unlikely, near the two vertical edges, field distribution is very low.

### Experimental result and discussion

**Figure 19:**
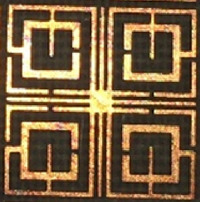
Fabricated MTM unit cell.

**Figure 20:**
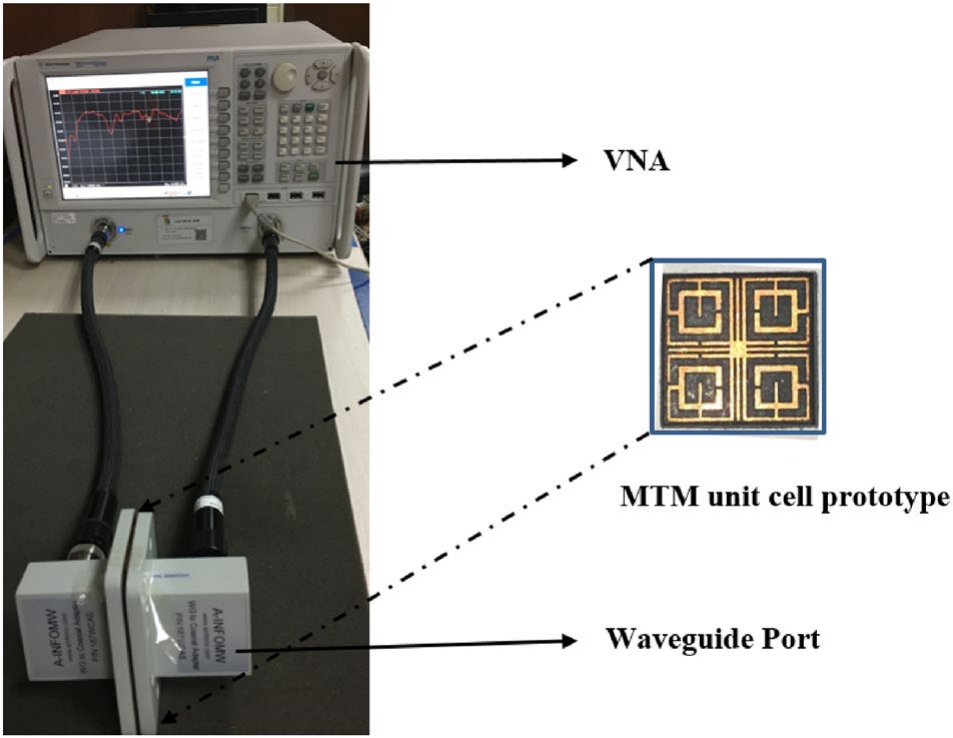
Experimental setup for measuring S_21_ for proposed MTM.

**Figure 21:**
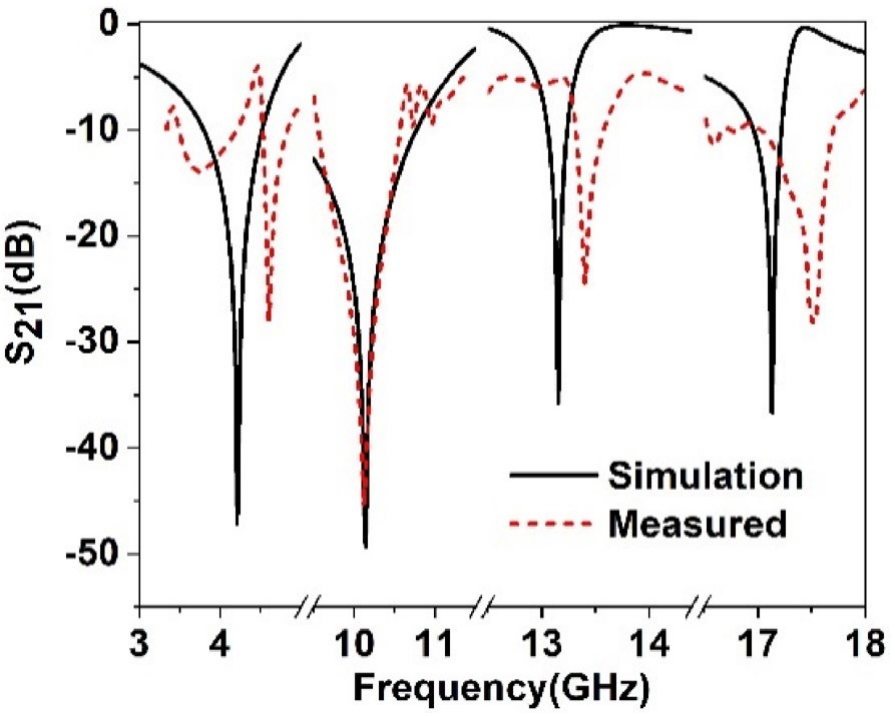
Measured transmission coefficient (S_21_) of the proposed unit cell.

The performance of the proposed MTM unit cell is verified by measuring the S_21_ by using a vector network analyzer (VNA). The fabricated MTM unit cell is depicted in Fig. 19, whereas the measurement setup is presented in Fig. 20. The prototype of the MTM unit cell is placed in between two waveguide ports, as shown in Fig. 20. The measured result is plotted in Fig. 21 along with the simulated result. As shown in Fig. 21, the measured result also exhibits multiple resonances covering C, X and Ku-bands. The measured result shows four resonances of S_21_ around 4.6 GHz, 10.12 GHz, 13.3 GHz, and 17.5 GHz with a negative peak of − 27 dB, − 45 dB, − 24 dB, − 28 dB. Comparing with the simulation, the deviations in resonance frequencies are 9.5%, 0.2%, 1.5%, and 2.4% at frequencies of 4.2 GHz, 10.14 GHz, 13.15 GHz and 17.1 GHz, respectively. A mismatch is also noticed in the magnitude between measured and simulation results. The disagreement of amplitude between measurement and simulation is nearly 40%, 8%, 31.8% and 23% for resonances at 4.2 GHz, 10.14 GHz, 13.15 GHz and 17.1 GHz. The measured result shows the bandwidth of 0.28 GHz, 1 GHz, 0.24 GHz and 0.65 GHz. In comparison with simulated bandwidth, it is observed that measured bandwidth is less at 4.2 GHz and 10.14 GHz, whereas bandwidths at the upper two resonances are higher.

Moreover, some amount of noise and harmonics are also observed in the measured result. A number of factors are involved in these discrepancies between measured and simulation results. Fabrication tolerance and error in fabrication in the prototyping of miniaturized slots and metal strips may cause a slight change in structure compared ideal case in simulation. Moreover, coupling effect of the waveguide ports is also added along with the cable loss. The calibration error associated with VNA is also included with the measurement. The combined effect of all these factors results in a mismatch of resonance frequencies, magnitude of S_21_ and bandwidth. Regardless of these, the mismatching error is not acute, and the experimental outcome displays a similar response pattern within the C, X and Ku bands.

### Comparison

The proposed MTM is compared with some recently published state of arts that is presented in Table 5, where the physical and electrical dimensions of the MTMs, resonance frequencies of transmission coefficients, covering band and EMR are considered major parameters for comparison. The effective medium ratio (EMR) is calculated by using the relation, ${\text{EMR}} = \frac{\lambda }{L}$ where λ is the wavelength calculated at the lowest resonance frequency and $L$ is the highest dimension of the MTM. From Table 5, it is observed that dimensions of the MTM unit cell of Ref.^19,20,25^ are less compared to the proposed MTM dimensions. Ref.^19,20^ also shows high EMR representing the compactness of these designs, but the number of resonances of them is less compared to our proposed design. MTM of Ref.^25^ covers Ku and K bands with an EMR of 4.4, which is much less compared to our proposed MTM. Though above mentioned MTMs have some superiority compared to our proposed design, but one limitation is that all these MTM are designed on FR-4 substrate. A critical constraint of FR-4 is that its loss tangent is high and at high frequencies performances are degraded and energy loss becomes high through this substrate material. On the other hand, proposed MTM is designed on Rogers (RT-5880) substrate which is composed of glass microfiber reinforce PTFE with a low dissipation factor. So, the proposed MTM is suitable for high-frequency applications. Ref.^24^ shows high EMR compared to our proposed MTM, but it only operates on S and X bands. Comparing with the rest of the state of arts in Table 5, the proposed MTM reveals its superiority in terms of physical and electrical dimensions, number of resonances, covering bands. Moreover, one important feature of the proposed MTM is its tunable capability for frequency selectivity that provides flexibility to adjust operating frequencies especially in X and Ku bands.

**Table 5:** Performance comparison of the proposed MTM with other states of arts (comparison is made based on the physical and electrical dimensions, resonance frequency, covering band and EMR).

References	Year	Dimension physical (mm × mm) electrical (λ × λ)	Resonance frequency (GHz)	Frequency band	EMR	Tunable property
^18^	2016	35 × 35 0.21 λ × 0.21λ	1.8	L	4.76	No
^19^	2020	9 × 9 0.125λ × 0.125λ	4.15, 10.84, 14.93	C, X and Ku	8.03	No
^20^	2019	5 × 5 0.125λ × 0.125λ	7.5	C	8	No
^24^	2020	10 × 10 0.12λ × 0.12λ	3.57,11.6	S and X	8.4	No
^25^	2017	5 × 5 0.23λ × 0.23λ	13.9, 27.5	Ku and K	4.4	No
^49^	2018	20 × 20 0.77λ × 0.77λ	11.5,13.5	X and Ku	1.4	No
^50^	2017	25 × 25 0.23λ × 0.23λ	2.8	S	4.28	No
^51^	2021	10.3 × 10.3 0.59λ × 0.59λ	17.1	Ku	1.7	No
Proposed	2021	10 × 10 0.14λ × 0.14λ	4.2, 10.14, 13.15, 17.1	C, X, Ku	7.17	Yes

As presented in Table 6, a comparative study is performed on the proposed MTM and some recently published tunable metamaterials based on the tuning mechanism and their effect on the responses of the particular MTM. In^52^, an external magnetic field is exerted by using very thin magnetic microwires that are imposed on the metamaterial along with the incident electromagnetic waves. By controlling the magnetic field of these wires by dc voltage, the resonance frequency can be controlled. On the other hand, in^53^, mercury inspired split ring resonator is described that exhibits magnetic resonance depending on temperature variation. Moreover, a tunable metamaterial filter is presented in^54^ that uses two cross coupled split rings. Two stopband frequencies are tuned by changing the angle between these two rings. In^55^, the bias voltage applied at two ends of a PN diode of GaAs placed over a metamaterial unit cell helps to tune the resonance frequency and magnitude of resonance at the THz range. Additionally, in^56^, electrical tuning is used to change the wavelength of the reflection spectra using metamaterial based on nanopillars of Ge and Al doped ZnO composition. Here, optical frequencies are controlled by the gate voltage, and phase modulation is achieved by nanopillars height modification. Comparing to these tuning metamaterials, our proposed MTM provides a more straightforward means to control the resonances of transmission coefficients and provides multiple resonances covering multiple bands extending from 4 GHz to 18 GHz. Moreover, the tunable frequency range is more extended in GHz frequencies compared to the other state of arts presented in Table 6.

**Table 6:** A comparative study of proposed MTM with some recently published tunable MTMs.

References	Tuning mechanism	Frequency/wavelength shifting	Covering band	Other features
^52^	CoFeSi-based magnetic microwire biased with DC voltage	0.2 GHZ	S-band	1. 100 wires are used 2. Thickness of wire 33 µm 3. Shift of frequency is 0.5% 4. Tuned resonance frequency is around 3.5 GHz
^53^	Mercury based toroidal resonator tuned by temperature change	7.2 MHz/°C	S-band	1. Resonance frequency shifts to low frequency with increasing temperature 2. Linear shift noticed within 0–30 °C
^54^	Tunability based on coupling between two crossed split ring resonators arranged in different rotation angles	2.5 GHz	C-band	1. Dual band resonances within 5.5–8 GHz 2. 2.5 GHz variation obtained within rotation angle 30°–90°
^55^	The potential difference between p-type GaAs and n-type GaAs over the unit cell controls the resonances	–	2–6 THz	1. Semiconductor-based metamaterial 2. Resonance frequencies and their magnitude are tuned by controlling free electrons concentration of unit cell
^56^	Electrical tuning by varying gate voltage of CMOS compatible nanopillars based metamaterial	240 nm	–	1. Wavelength shifting obtained for voltage change from – 4 V to + 4 V 2. More than 40% differential reflection is experimentally observed 3. Phase modulation up to 270° is achieved by optimizing nanopillar heights
Proposed	Tunability based on changing the length of four metallic stubs extended from the center and placed between four symmetrical quartiles	110 MHz 1.12 GHz 3.1 GHz	C-band X-band Ku-band	1. Symmetrical split-ring resonators are used 2. Frequency shifts are more pronounced within 12–18 GHz and less within 4–8 GHz 3. Simultaneous change of four tuning stubs from 2.5 to 5 mm helps to adjust the resonance frequencies

## Conclusion

A tuning metamaterial is presented in this article that exhibits quad resonances of S_21_ covering C, X and Ku bands. The MTM unit cell is designed on Rogers (RT5880) substrate having a thickness of 1.57 mm and electrical dimension 0.14λ × 0.14λ. The patch of the resonator is symmetric in design which contains four quartiles of equal and similar shape made with interconnecting two split-ring resonators. A square metal strip at the center interconnects four quartiles, and four tuning metal strips are attached to it and placed as a spacer between four quartiles. The unit cell provides resonances of S_21_ at 4.2 GHz, 10.14 GHz, 13.15 GHz and 17.1 GHz. It also displays negative permittivity, near zero permeability, near zero refractive index and a high EMR of 7.17. A unique property of the proposed unit cell is that its resonance frequency can be tuned by changing the length of the tuning metal stubs with the flexibility of robust adjustment resonance frequency in X and Ku bands. The equivalent circuit model of the MTM is performed and justified by comparing S_21_ in ADS with S_21_ of CST with good agreement. Surface current, electric and magnetic fields are analyzed. An experiment is performed to determine S_21_ that shows good similarity with the simulation results. High EMR, negative permittivity, near zero permeability and refractive index, and frequency selective capability through tuning make this MTM suitable for applications in microwave devices covering C, X and Ku bands.
